# Hepatocyte BPGM Induces RET Lactylation and Macrophage Reprogramming to Promote Tumorigenesis in Hepatocellular Carcinoma

**DOI:** 10.1002/advs.202518180

**Published:** 2026-01-09

**Authors:** Jiajia Zhang, Lu Shi, Liangliang Lin, Yang Zhang, Miao Zhang, Lili Wu, Yong Xia, Yaqiong Zhang, Peng Han, Likun Zhuang, Liang Shi

**Affiliations:** ^1^ Department of Laboratory Medicine The Eighth Affiliated Hospital of Sun Yat‐Sen University Shenzhen P. R. China; ^2^ Central Laboratories Qingdao Municipal Hospital Qingdao University Qingdao P. R. China; ^3^ Department of Clinical Transfusion The Eighth Affiliated Hospital of Sun Yat‐Sen University Shenzhen Guangdong P. R. China; ^4^ Department of Clinical Medical Laboratory Peking University Shenzhen Hospital Shenzhen Guangdong P. R. China; ^5^ Department of Clinical Laboratory Taizhou Key Laboratory of Research and Transformation of Extracellular Vesicles Taizhou Central Hospital (Taizhou University Hospital) Zhejiang Taizhou P. R. China; ^6^ Genomics and Big Data Institute Research and Innovation Center, Evomics Qingdao P. R. China

**Keywords:** BPGM, HCC, macrophage polarization, protein lactylation, RET

## Abstract

Aerobic glycolysis is a hallmark of cancer, yet the role of the key glycolytic enzyme bisphosphoglycerate mutase (BPGM) in hepatocellular carcinoma (HCC) progression remains unclear. Here, clinical sample analyses revealed that BPGM expression was upregulated in HCC tissues and associated with poor prognosis. Hepatocyte‐specific *Bpgm* knockout significantly attenuated DEN‐induced HCC development in mice. Spatial transcriptomics and single‐cell RNA sequencing revealed that hepatocyte‐specific *Bpgm* knockout reduced the monocyte/macrophage infiltration and decreased M2 polarization of tumor‐associated macrophages. Additionally, *BPGM* overexpression promoted the proliferation and migration of HCC cells and enhanced intracellular lactate accumulation. Liquid chromatography‐tandem mass spectrometry (LC‐MS/MS) identified ret proto‐oncogene (RET) as a downstream effector that mediated the effects of BPGM on HCC cells. BPGM promoted P300‐mediated lactylation of RET at lysine 549 (K549), which competitively inhibited its ubiquitination, thereby preventing RET protein degradation and enhancing its stability. BPGM in HCC cells also induced both histone lactylation and M2 polarization of macrophages by lactate secretion. This study revealed that BPGM in hepatocytes could enhance RET expression via increasing its lactylation in malignant cells and promote M2 polarization of macrophages, both of which contributed to HCC progression. These findings established that BPGM could act as a potential therapeutic target for HCC.

## Graphical abstract

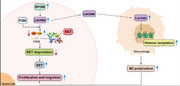

**Abbreviations**
ANOVA: one‐way analysis of variance
BPGM: bisphosphoglycerate mutase
CCK‐8: cell counting kit 8
CHC: Alpha‐cyano‐4‐hidroxycinnamate
CON: control
DMEM: Dulbecco's modified Eagle medium
DMSO: dimethyl sulfoxide
FBS: fetal bovine serum
GEO: Gene Expression Omnibus
GDNF: glial cell line‐derived neurotrophic factor
GSEA: gene set enrichment analysis
HCC: hepatocellular carcinoma
HPA: Human Protein Atlas
IF: immunofluoresence
IP: immunoprecipitation
LC‐MS/MS: liquid chromatography‐tandem mass spectrometry
siRNA: small interfering RNA
PTMs: protein posttranslational modifications
TAMs: tumor‐associated macrophages
TCGA: The Cancer Genome Atlas
TME: tumor microenvironment
OE: overexpression
PMA: phorbol 12‐myristate 13‐acetate
RET: ret proto‐oncogene
scRNA‐seq: single cell RNA sequencing
SD: standard deviation
SDS: sodium dodecyl sulfate
Slc2a1: solute carrier family 2 member 1
UMAP: Uniform Manifold Approximation and Projection

## 1 Introduction

Hepatocellular carcinoma (HCC), which constitutes the major histological subtype of liver cancer, poses significant therapeutic challenges and ranks sixth in global incidence and third in terms of cancer‐related mortality worldwide [1, 2]. HCC is highly malignant due to its insidious onset, rapid progression, and metastasis. Therefore, elucidating the molecular pathogenesis of HCC and discovering more specific anticancer therapeutic targets is urgent.

Metabolic reprogramming is a key feature of cancers, including HCC, that enables tumor cells to acquire metabolic adaptations to support continued proliferation. Warburg effect, the most widely known feature of metabolic reprogramming, is a phenomenon in which tumor cells preferentially metabolize glucose via aerobic glycolysis rather than mitochondrial oxidative phosphorylation, even under normoxic conditions [3, 4]. Consequently, aerobic glycolysis leads to high glucose consumption and lactate accumulation.

Recently, lactate has been found to modulate nuclear histones by adding a lactyl group to the lysine (K) residue of histones [5]. This process, termed “lactate‐derived lysine lactylation (Kla)”, has been identified as a new metabolite‐induced posttranslational modification (PTM). Histone lactylation regulates gene expression in both tumor cells and immune cells, thereby modulating tumor progression and immunosuppression [6, 7, 8]. Non‐histone protein lactylation has also been implicated in regulating critical oncogenic processes, including tumor proliferation and drug resistance [9, 10]. In addition, the large amount of lactate produced by tumors creates a low‐nutrient, hypoxic, and low pH tumor microenvironment (TME). This affects immune cells in various ways, including suppressing the function of T cells and natural killer (NK) cells, and promoting the recruitment of regulatory T cells to drive immune escape [11]. Studies have demonstrated that tumor‐derived lactate induces macrophage polarization towards the M2 phenotype via G protein‐coupled receptor (GPR)‐ and monocarboxylate transporter protein (MCT)‐mediated “lactate shuttling”, fostering an immunosuppressive microenvironment in tumors [12, 13, 14, 15]. Therefore, therapeutic strategies targeting lactate are a promising new therapeutic approach for treating HCC.

Bisphosphoglycerate mutase (BPGM), a glycolysis‐associated enzyme, catalyzes the conversion of the glycolytic intermediate 1,3‐diphosphoglycerate (1,3‐BPG) to 2,3‐diphosphoglycerate (2,3‐BPG). By regulating glycolytic intermediate levels, BPGM mediates serine biosynthesis flux, a critical process for macromolecular biosynthesis that supports rapid cancer cell proliferation [16]. Notably, studies on HCC pathogenesis have demonstrated progressively elevated BPGM expression during the transition from viral hepatitis to HCC, with its overexpression correlated significantly with adverse clinical outcomes [17].

Our study aimed to comprehensively investigate the role of BPGM in the progression of HCC by utilizing hepatocyte‐specific *Bpgm*‐knockout (*Bpgm*‐CKO) mice, spatial transcriptomics, and single‐cell RNA sequencing (scRNA‐seq). We also aimed to elucidate the mechanism through which BPGM modulates ret proto‐oncogene (RET) expression, leading to increased proliferation and migration ability of HCC cells. We identified that BPGM regulates the lactylation and ubiquitination of *RET*‐K549 to alter the stability of the RET protein. We further validated the significant role of BPGM in promoting the M2 polarization of macrophages. Through this study, we provide new scientific insight into the mechanism underlying the pathogenesis of HCC and explore novel biomarkers for HCC patients.

## 2 Results

### 2.1 BPGM is Upregulated in Tumor Tissues and Associated with Poor Prognosis of HCC Patients

**FIGURE 1:**
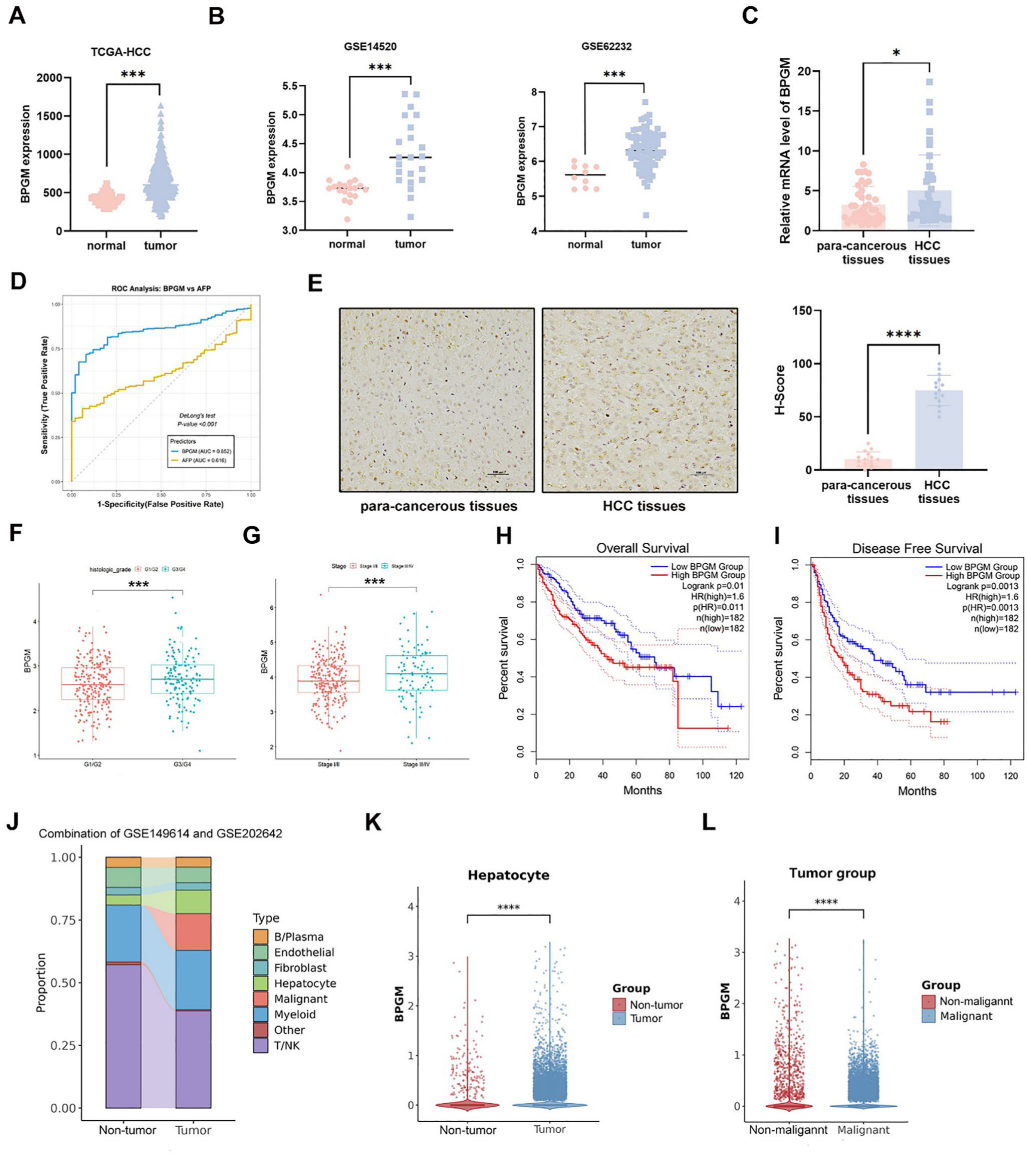
The expression level of BPGM is upregulated in HCC tissues. (A) *BPGM* mRNA expression in the TCGA HCC cohort. (B) *BPGM* mRNA expression in HCC in the GEO database. (C) qRT‐PCR analysis of *BPGM* mRNA expression in 40 HCC tissues and para‐cancerous tissues (*n* = 40). (D) Analysis of the diagnostic efficacy of *BPGM* and AFP for HCC. (E) Protein levels of BPGM in fifteen pairs of HCC tissues and para‐cancerous tissues were measured by IHC staining (*n* = 15). (F) *BPGM* expression in HCC patients with different histologic grades. (G) *BPGM* expression in HCC patients with different stages. (H, I) Correlation between *BPGM* expression and overall survival (H) or disease‐free survival (I). (J) Proportions of cell types in non‐tumor and tumor groups from single‐cell RNA sequencing analysis. (K) *BPGM* expression in non‐tumor and tumor groups of hepatocytes from single‐cell RNA sequencing analysis. (L) *BPGM* expression in non‐malignant and malignant HCC cells from the tumor group from single‐cell RNA sequencing analysis. *p*‐Values were calculated using a two‐tailed Student's *t*‐test. The data were presented as the mean±SD. ns, no significance; ^*^, *p* < 0.05; ^**^, *p* < 0.01; ^***^, *p* < 0.001; ^****^, *p* < 0.0001.

We first interrogated The Cancer Genome Atlas (TCGA) and Gene Expression Omnibus (GEO) databases to assess *BPGM* transcriptional levels during HCC progression, finding that *BPGM* mRNA expression was significantly elevated in tumor tissues from HCC patients compared to normal liver tissues (*p* < 0.001) (Figure 1A,B). Consistently, the results from our cohort also showed significantly higher mRNA levels of *BPGM* in tumor tissues than para‐cancerous tissues of HCC patients (*n* = 40) (*p* < 0.05) (Figure 1C). Based on the TCGA dataset, DeLong's test for correlated Receiver Operating Characteristic (ROC) curves revealed that the area under the curve (AUC) of *BPGM* (0.8517) was significantly higher than that of *AFP* (0.6160) in distinguishing HCC tissues from normal liver tissues (*p* < 0.001), confirming a statistically significant superiority of BPGM over AFP in diagnostic efficacy (Figure 1D). Furthermore, we performed IHC staining on HCC tissues and para‐cancerous tissues (*n* = 15) to examine the protein expression of BPGM in HCC patients, and found higher expression of BPGM in HCC tissues (*p* < 0.0001) (Figure 1E). Analysis of the TCGA dataset revealed that patients with high tumor grade (G3/G4) and advanced stage (III/IV) showed elevated *BPGM* mRNA levels (*p* < 0.05) (Figure 1F,G). Then, we correlated the expression of *BPGM* and the survival rate of HCC patients based on the TCGA database, and identified that patients with high expression of *BPGM* exhibited a shorter overall survival (OS) and disease‐free survival (DFS) (Figure 1H,I). These data confirmed that BPGM was overexpressed in HCC tissues at both transcriptional and protein levels, which is associated with poor patient prognosis, suggesting the potential of BPGM as a diagnostic biomarker for HCC.

To investigate the expression of BPGM in different cell types, we performed analysis for the integrated scRNA‐seq data of HCC samples from two datasets, GSE149614 (8 non‐tumor and 10 tumor samples) and GSE202642 (4 non‐tumor and 7 tumor samples). Inferred copy number variation (CNV) analysis validated the presence of tumor cells. The cell proportions were shown in Figure 1J. We observed that the expression of *BPGM* was significantly higher in hepatocytes in tumor tissues than that in non‐tumor liver tissues (*p* < 0.0001) (Figure 1K). While in tumor tissues, *BPGM* expression levels were significantly higher in malignant HCC cells than in non‐malignant hepatocytes (*p* < 0.0001) (Figure 1L). In addition, the expression levels of *BPGM* in endothelial cells, fibroblasts, and B/plasma cells in tumor tissues were also significantly increased than those in non‐tumor liver tissues (Figure S1A–C), while this difference was not observed in myeloid cells and T/NK cells (Figure S1D,E). These results suggested that hepatocyte‐specific BPGM might function as a tumor‐promoting factor in HCC pathogenesis.

### 2.2 Knocking out BPGM Inhibits HCC Tumor Growth In Vivo

**FIGURE 2:**
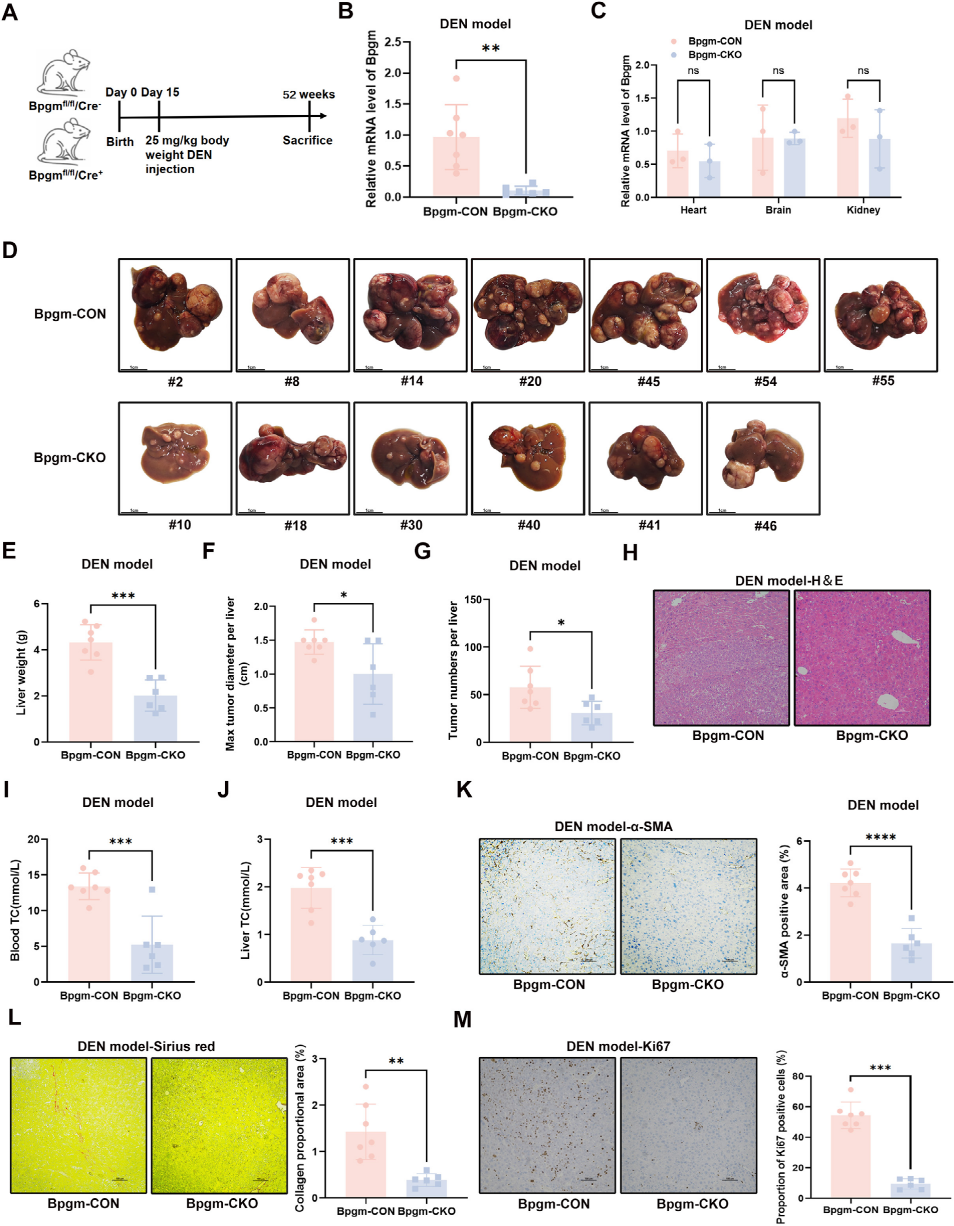
Knockout of BPGM inhibits HCC tumor growth in vivo. (A) Experimental schematic of DEN‐treated mice (*Bpgm* ^fl/fl^ *Cre* ^−^, *n* = 7; *Bpgm* ^fl/fl^ *Cre* ^+^, *n* = 6). (B,C) qRT‐PCR analysis of *Bpgm* expression in liver (*Bpgm*‐CON, *n* = 7; *Bpgm*‐CKO, *n* = 6), heart, brain, and kidney (*Bpgm*‐CON, *n* = 3; *Bpgm*‐CKO, *n* = 3). (D) Images of livers from *Bpgm*‐CON and *Bpgm*‐CKO mice (*Bpgm*‐CON, *n* = 7; *Bpgm*‐CKO, *n* = 6). (E–G) Liver weight, max tumor diameter, and tumor numbers in the *Bpgm*‐CON and *Bpgm*‐CKO mice (*Bpgm*‐CON, *n* = 7; *Bpgm*‐CKO, *n* = 6). (J) H&E staining of mouse livers in the *Bpgm*‐CON and *Bpgm*‐CKO mice. (I, J) The level of TC in the blood and liver of *Bpgm*‐CON and *Bpgm*‐CKO mice (*Bpgm*‐CON, *n* = 7; *Bpgm*‐CKO, *n* = 6). (K–M) IHC staining of α‐SMA, Sirius red staining, and IHC staining of Ki‐67 in liver tissues from the *Bpgm*‐CON and *Bpgm*‐CKO mice (*Bpgm*‐CON, *n* = 7; *Bpgm*‐CKO, *n* = 6). Differences between the two groups were assessed using the two‐tailed Student's *t*‐test. ns, no significance; ^*^, *p* < 0.05; ^**^, *p* < 0.01; ^***^, *p* < 0.001; ^****^, *p* < 0.0001.

To analyze the role of BPGM in hepatocarcinogenesis, CRISPR‐Cas9 and Loxp‐*Cre* technologies were used to construct a hepatocyte‐specific *Bpgm*‐knockout mouse model (Figure S2A–C). The HCC mouse model was induced with an intraperitoneal injection of the alkylating agent diethylnitrosamine (DEN) (Figure 2A). Compared with *Bpgm*‐CON mice, *Bpgm*‐CKO mice exhibited significantly reduced *Bpgm* mRNA expression in the liver (*p* < 0.01), with no differences detected in the heart, brain, or kidney (Figure 2B,C). These results confirmed the successful construction of a hepatocyte‐specific *Bpgm*‐knockout mouse model. Numerous nodules of different sizes, occupying most of the liver surface area, were visible in the *Bpgm*‐CON mice. In the *Bpgm*‐CKO mice, the livers were almost similar in appearance, color, and stiffness to normal livers, except for a few nodules on the liver surface (Figure 2D). In total, liver weight (*p* < 0.001), the largest tumor diameter (*p* < 0.05), and tumor number (*p* < 0.05) were all significantly decreased in the *Bpgm*‐CKO mice compared with the *Bpgm*‐CON mice (Figure 2E–G). Hematoxylin‐eosin (H&E) staining of the liver further confirmed the morphological features of HCC in the *Bpgm*‐CON mice (Figure 2H). Additionally, knocking out *Bpgm* significantly reduced the serum and hepatic levels of total cholesterol (TC) (*p* < 0.001) (Figure 2I,J). Compared with the *Bpgm*‐CKO mice, the *Bpgm*‐CON mice exhibited a higher degree of fibrosis as demonstrated by immunohistochemical (IHC) staining of alpha‐smooth muscle actin (α‐SMA) (*p* < 0.0001) and Sirius red staining (*p* < 0.01) (Figure 2K,L). IHC staining of Ki‐67 revealed a significantly higher proliferative capacity in the *Bpgm*‐CON mice than that in the *Bpgm*‐CKO mice (*p* < 0.001) (Figure 2M). Together, hepatocyte‐specific BPGM deficiency attenuated HCC progression, suggesting that BPGM may participate in hepatocarcinogenesis.

### 2.3 BPGM Expression Notably Enhances the Infiltration of Monocytes/Macrophages into the HCC TME

**FIGURE 3:**
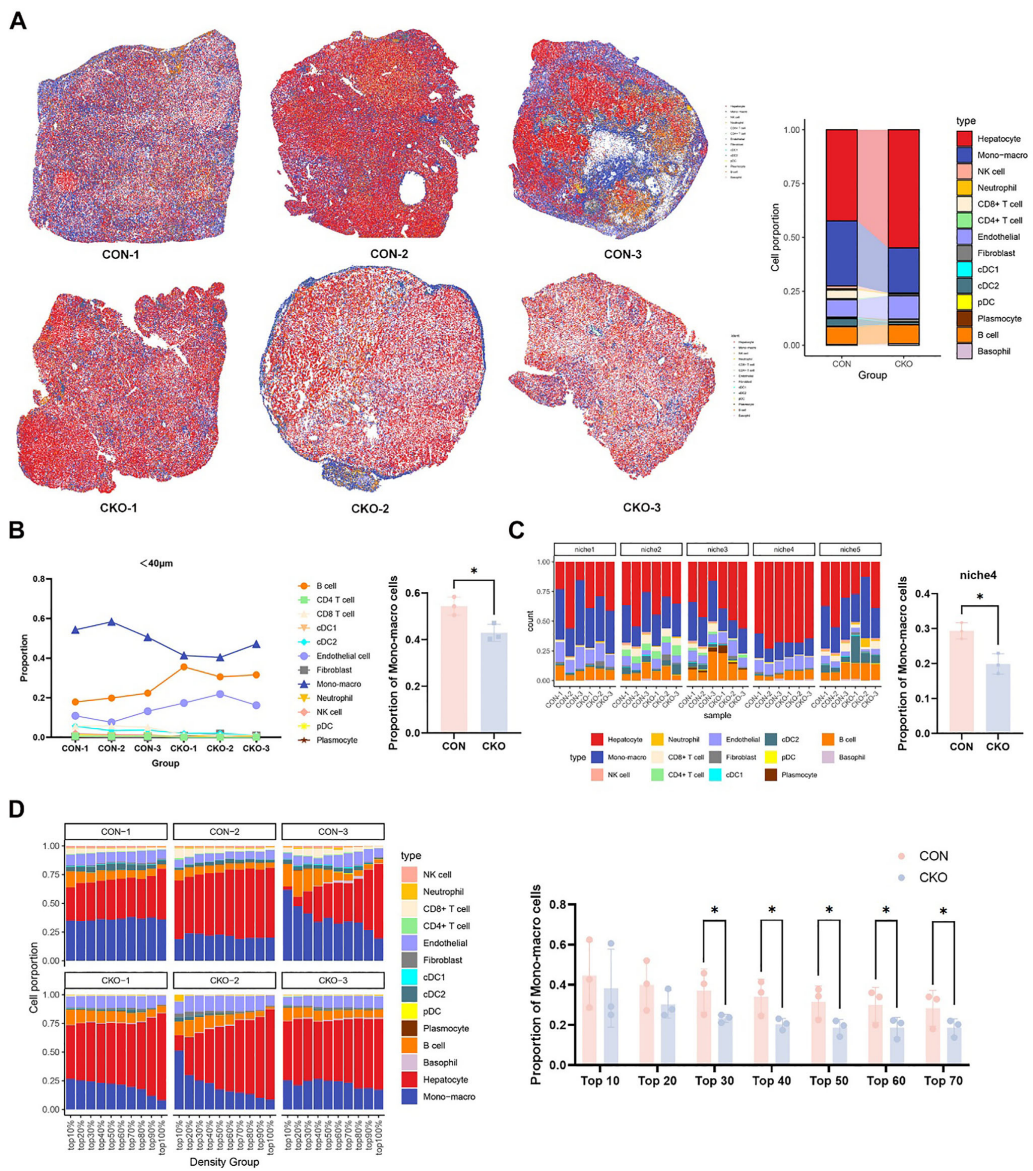
Spatial distribution of monocytes/ macrophages in the HCC TME. (A) Spatial distribution (left) and proportion (right) of different cell types. (B) Analysis of cellular proportions within 40 µm of hepatocytes. (C) Spatial niche analysis of different cell types (*k* = 5; niche 1 to niche 5). (D) The proportions of monocytes/ macrophages in the *Bpgm*‐CON and *Bpgm*‐CKO mice within the top 10% to top 70% density regions of hepatocytes. The data are presented as the means ± SDs. *N* = 3 in the *Bpgm*‐CON and *n* = 3 in the *Bpgm*‐CKO group. *p‐*Values were calculated using a two‐tailed Student's *t*‐test. ^*^, *p* < 0.05.

To comprehensively analyze the spatial distribution profile of different cell types within HCC tumors, we collected six tumor tissue specimens from the *Bpgm*‐CON and *Bpgm*‐CKO mice with HCC (*n* = 3 per group) and utilized Seurat to integrate scRNA‐seq with spatial transcriptome data, thereby reconstructing a spatial single cell map (Figure 3A). We further found that monocytes/ macrophages were the most abundant cell type within a 40 µm radius of hepatocytes from *Bpgm*‐CON mice (*p* < 0.05) (Figure 3B). Spatial niche analysis revealed that the proportion of monocytes/ macrophages in hepatocyte‐enriched niche 4 was significantly higher in the *Bpgm*‐CON mice than that in the *Bpgm*‐CKO mice (*p* < 0.05) (Figure 3C). Following hepatocyte density stratification, we found that the proportion of monocytes/ macrophages was significantly higher in the *Bpgm*‐CON mice than the *Bpgm*‐CKO mice in regions corresponding to a hepatocyte density of top 30–70% (*p* < 0.05) (Figure 3D; Figure S3). Collectively, we identified that BPGM expression promotes monocyte/ macrophage infiltration within the TME of HCC.

### 2.4 BPGM Induces M2 Polarization of TAMs at the Single‐Cell Level

**FIGURE 4:**
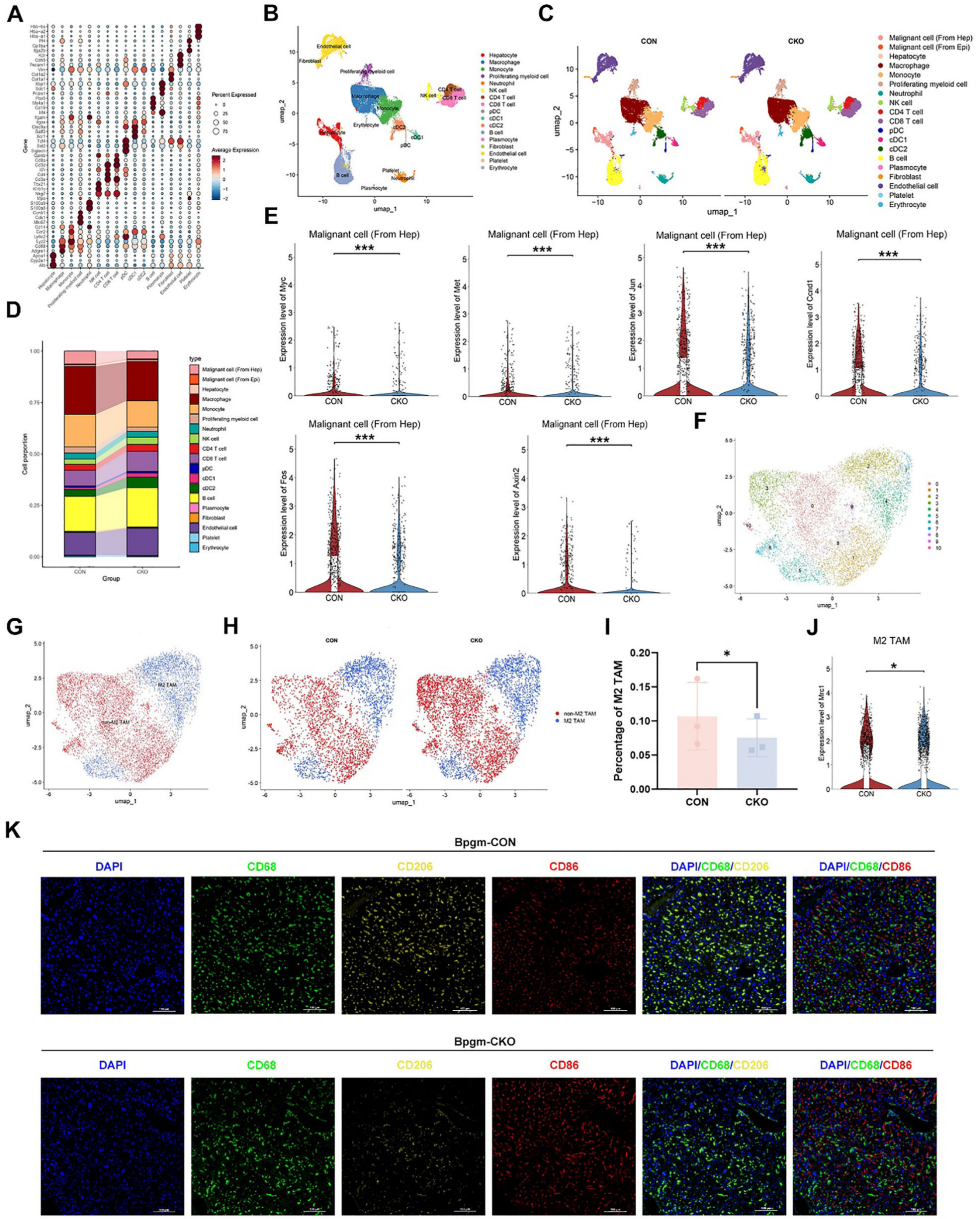
Single cell sequencing analysis of the *Bpgm*‐CON (*n* = 3) and *Bpgm*‐CKO (*n* = 3) mice with HCC. (A) Marker gene dot plot. (B) UMAP of cell clusters identified from the scRNA‐seq data. (C) UMAP of cell clusters identified from the scRNA‐seq data of three tissues from the *Bpgm*‐CON mice (left) and three tissues from the *Bpgm*‐CKO mice (right). (D) Analysis of cell proportions in the *Bpgm*‐CON and *Bpgm*‐CKO mice. (E) Expression levels of *Myc*, *Met*, *Jun*, *Ccnd1*, *Fos*, and *Axin2* genes in malignant hepatocytes from the *Bpgm*‐CON and *Bpgm*‐CKO mice. (F) UMAP plot of 11 cell clusters. (G) UMAP plot of the M2 TAM group and the non‐M2 TAM group. (H) UMAP plot of M2 TAM cells and non‐M2 TAM cells in the *Bpgm*‐CON and *Bpgm*‐CKO mice. (I) Percentage of M2 TAMs in the *Bpgm*‐CON and *Bpgm*‐CKO mice (*n* = 3). (J) Expression level of the M2 TAM marker gene *Mrc1* in M2 TAMs from the *Bpgm*‐CON and *Bpgm*‐CKO mice. (K) The liver tissues of *Bpgm*‐CON and *Bpgm*‐CKO groups were subjected to immunofluorescent staining of CD68 (green), CD206 (yellow), and CD86 (red). Nuclei were stained with DAPI (blue). The data are presented as the means ± SDs. Two‐tailed Student's *t*‐test was used to evaluate statistical significance. ^*^, *p* < 0.05; ^***^, *p* < 0.001.

To assess the plasticity and phenotypes of the immune cells within the HCC microenvironment at the single‐cell level, we performed scRNA‐seq on tumor tissues from the *Bpgm*‐CON and *Bpgm*‐CKO mice. We categorized the cell populations into 17 clusters based on enrichment analysis of differentially expressed genes (DEGs) (Figure 4A). The Uniform Manifold Approximation and Projection (UMAP) profiles of the *Bpgm*‐CON and *Bpgm*‐CKO mice were shown in Figure 4B and Figure 4C. Proportion analysis revealed reduced percentages of malignant cells (derived from hepatocytes) and macrophages in the *Bpgm*‐CKO mice (Figure 4D). Furthermore, proliferation pathway effectors, including *Myc* [18], *Met* [19], *Jun* [20], *Ccnd1* [21], *Fos* [22], and *Axin2* [23], were highly expressed in malignant hepatocytes from the *Bpgm*‐CON mice (*p* < 0.001) (Figure 4E). Subsequently, we performed a subclustering analysis on the macrophages, identifying 11 distinct subsets (Figure 4F). Using the M2 marker *Mrc1*, we defined two major populations: M2‐polarized tumor‐associated macrophages (M2 TAMs) and non‐M2 TAMs (Figure 4G–H). Strikingly, compared to the *Bpgm*‐CKO mice, the *Bpgm*‐CON mice exhibited both significant expansion of the M2 TAM population and enhanced *Mrc1* expression in M2 TAM (*p* < 0.05) (Figure 4I–J). Similarly, the immunofluorescence results of liver tissues revealed the enhanced number of CD68+/CD206+ M2 macrophages in *Bpgm*‐CON mice (Figure 4K). Collectively, these findings demonstrated that BPGM in HCC cells drove the coordinated activation of malignant cells (derived from hepatocytes) and M2 TAMs.

### 2.5 BPGM Promotes the Proliferation and Migration of HCC Cells In Vitro

**FIGURE 5:**
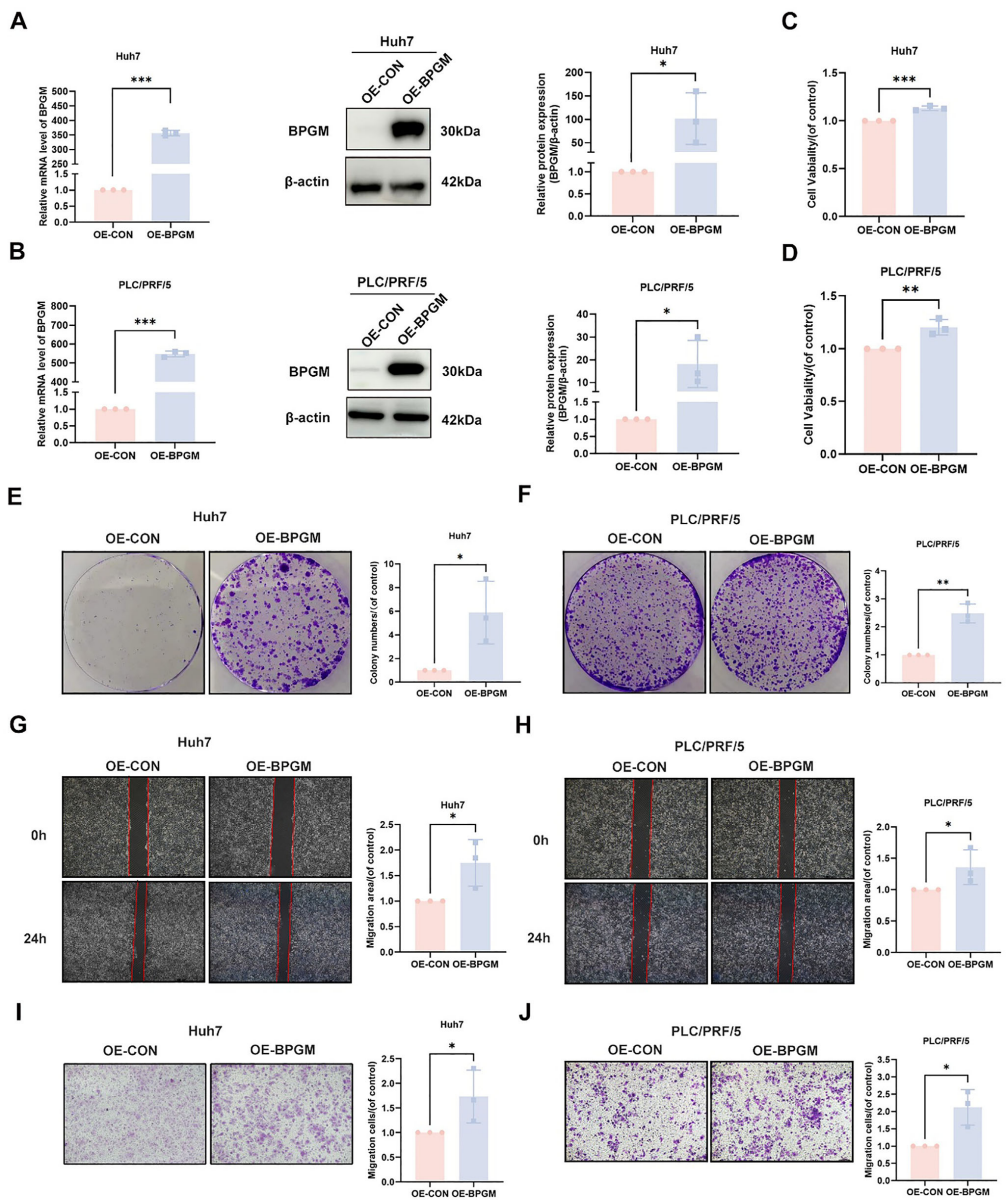
BPGM overexpression promotes HCC cell proliferation, migration, and colony formation abilities. (A, B) qRT‐PCR and western blot analysis of *BPGM* expression level in *BPGM*‐overexpressed Huh7 and PLC/PRF/5 cells (*n* = 3). (C–J) CCK‐8, colony formation, wound‐healing, and Transwell assays in *BPGM*‐overexpressed Huh7 and PLC/PRF/5 cells (*n* = 3). The data are presented as the means ± SDs. Two‐tailed Student's *t*‐test was used to evaluate statistical significance. ^*^, *p* < 0.05; ^**^, *p* < 0.01; ^***^, *p* < 0.001.

Next, we generated the *BPGM*‐overexpressed HCC cell lines Huh7 and PLC/PRF/5 to investigate the functional role of BPGM. Successful overexpression of *BPGM* was verified at both the RNA and protein levels (Figure 5A,B). The results of the CCK‐8 assay and colony formation assay indicated that the overexpression of *BPGM* significantly enhanced the proliferation rates of Huh7 and PLC/PRF/5 cells (Figure 5C–F). Wound‐healing and Transwell assays demonstrated that the overexpression of *BPGM* significantly enhanced the migratory capacity of HCC cells (*p* < 0.05) (Figure 5G–J). Furthermore, we used *BPGM*‐specific siRNA to knock down gene expression in the HCC cell lines (Figure S4A,B). We found that *BPGM* knockdown with si‐*BPGM*‐3 significantly reduced the proliferation, migration, and colony‐forming capacities of HCC cells (Figure S4C–J). *BPGM* knockdown with another siRNA (si‐*BPGM*‐2) targeting a different site also showed the same phenotypic changes (Figure S5A–C). Collectively, these findings demonstrated that targeting BPGM significantly affects the malignant phenotypes of the HCC cells, including proliferation and migration.

### 2.6 BPGM Correlates Positively with Lactate Production and Protein Lactylation in HCC Cells

**FIGURE 6:**
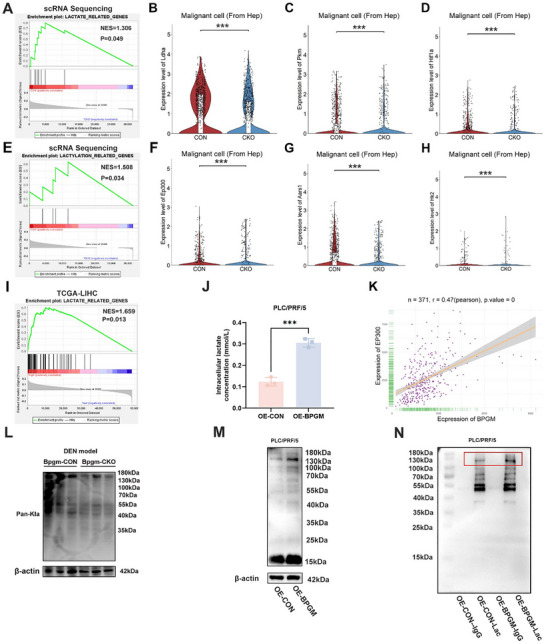
BPGM induces lactate production and lactylation of HCC cells. (A) GSEA of lactate‐related genes from mouse scRNA‐seq data. (B–D) Expression levels of *Ldha*, *Pkm*, and *Hif1α* in the *Bpgm*‐CON and *Bpgm*‐CKO mice. (E) GSEA of lactylation‐related genes from mouse scRNA‐seq data. (F–H) Expression level of *Ep300*, *Aars1*, and *Hk2* in the *Bpgm*‐CON and *Bpgm*‐CKO mice. (I) GSEA of lactate‐related genes in the *BPGM* high‐expression group from the TCGA dataset. (J) Intracellular lactate concentration in the OE‐CON and OE‐*BPGM* groups (*n* = 3). (K) Correlation analysis of *BPGM* and *EP300* genes. (L) The level of pan‐lactylation in the *Bpgm*‐CON and *Bpgm*‐CKO mice. (M) The level of pan‐lactylation in the OE‐CON and OE‐*BPGM* groups was assessed using a Western blot. (N) Cells of the OE‐CON and OE‐*BPGM* groups were lysed and subjected to anti‐Pan Kla IP. A Western blot was performed to analyze differences in the bands. Differences between the two groups were assessed using a two‐tailed Student's *t*‐test. The data are presented as the means ± SDs. ^***^, *p* < 0.001.

Gene set enrichment analysis (GSEA) of transcriptome in malignant cells (from hepatocytes) from the *Bpgm*‐CON and *Bpgm*‐CKO mice revealed significant enrichment in biological processes of lactate metabolism (*p* = 0.049) and lactylation (*p* = 0.034) (Figure 6A,E). In addition, lactate‐associated genes (*Ldha*, *Pkm*, and *Hif1α*) were upregulated in malignant cells (from hepatocytes) in the *Bpgm*‐CON mice (*p* < 0.001) (Figure 6B–D). A similar finding was observed in lactylation‐related genes (*Ep300*, *Aars1*, and *Hk2*) (*p* < 0.001 or *p* < 0.05) (Figure 6F–H). The scRNA‐seq data from the GSE149614 dataset also demonstrated that hepatocytes in HCC tumor tissues exhibited higher *LDHA*, *PKM*, and *SLC2A1* levels (*p* < 0.0001) (Figure S6A–C). Consistently, lactate‐related signaling pathways were significantly enriched in the group of HCC patients with high *BPGM* expression from the TCGA database (Figure 6I). Given the notable upregulation of glycolysis in cancers and that lactate is a key metabolite of glycolysis, it was important to explore the involvement of the lactate‐related signal in HCC. In vitro experiments showed that a significant increase in intracellular lactate concentration was observed in *BPGM*‐overexpressed PLC/PRF/5 cells (*p* < 0.001) (Figure 6J). Furthermore, correlation analysis showed a significant positive correlation between the expression of *BPGM* and *EP300* in 371 tumor tissues of HCC patients from the TCGA database (r = 0.47, *p* < 0.0001) (Figure 6K). These results indicated that *BPGM* mRNA levels are closely related to lactate and lactylation. The level of pan‐lactylation in the livers of the *Bpgm*‐CKO mice was significantly decreased compared with the *Bpgm*‐CON mice (Figure 6L), while the intracellular levels of protein lactylation in *BPGM*‐overexpressed PLC/PRF/5 cells were also increased (Figure 6M). Furthermore, we performed IP experiments in *BPGM*‐overexpressed PLC/PRF/5 cells to enrich the downstream target proteins of BPGM‐mediated lactylation. After SDS‐PAGE of the immunoprecipitates, the gels were cut off and stained with silver nitrate. Western blot experiments on the immunoprecipitates showed that the level of lactylation was upregulated in the OE‐*BPGM*‐Lac group compared with the OE‐CON‐Lac group. The molecular weights of the differential proteins with lactylation were concentrated in the 130–180 kDa range, suggesting that BPGM might target non‐histone proteins within HCC cells (Figure 6N). These results suggested that BPGM facilitates lactate production and lactylation in HCC cells.

### 2.7 BPGM Regulates Lactylation and Ubiquitination of RET‐K549 in HCC Cells

**FIGURE 7:**
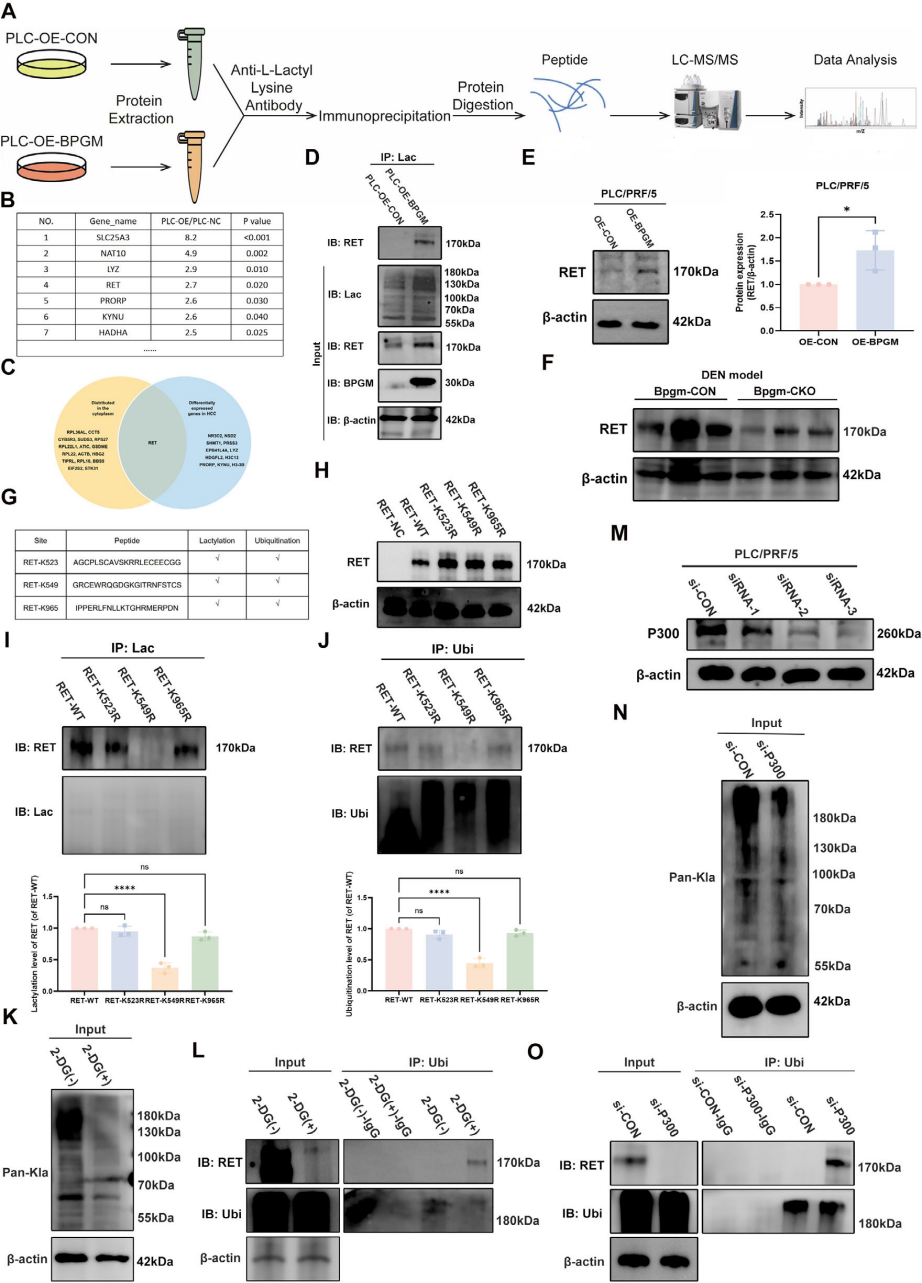
BPGM regulates lactylation and ubiquitination of *RET*‐K549 in HCC cells. (A) Workflow diagram for screening downstream proteins regulated by lactylation. (B) Partial proteins identified by LC‐MS/MS screening. (C) A Venn diagram showing overlapping hits from multiple screening conditions. (D) Co‐IP with anti‐lactylation antibody followed by western blot analysis of RET in lysates from PLC/PRF/5 OE‐CON and OE‐*BPGM* groups. (E) Western blot analysis to evaluate the expression level of RET in the OE‐CON and OE‐*BPGM* groups. (F) Western blot analysis to evaluate the expression level of RET in the *Bpgm*‐CON and *Bpgm*‐CKO mice. (G) Sequence information of the sites (K523, K549, and K965). (H) Western blot analysis to detect the overexpression efficiency of *RET*‐WT and the *RET* mutants (K523R, K549R, and K965R). (I, J) Co‐IP with anti‐lactylation or anti‐ubiquitination antibody followed by western blot analysis of PLC/PRF/5 cells transfected with *RET*‐WT or *RET* mutants (K523R, K549R, K965R). (K) Western blot analysis of Pan‐Kla expression levels in PLC/PRF/5 cells treated with or without 2‐DG. (L) Co‐IP with anti‐ubiquitination antibody followed by western blot analysis of 2‐DG‐treated or untreated PLC/PRF/5 cell lysates. (M) Western blot analysis of P300 expression in PLC/PRF/5 cells transfected with si‐CON or si‐*P300*. (N) Western blot detection of Pan‐Kla levels in si‐CON and si‐*P300* groups. (O) Co‐IP with anti‐ubiquitination antibody followed by Western blot analysis of PLC/PRF/5 cell lysates. The data are presented as the means ± SDs. Statistical significance was assessed by a two‐tailed Student's *t*‐test for two‐group comparisons and by one‐way ANOVA for multiple comparisons among four groups, respectively. ns, no significance; ^*^, *p* < 0.05; ^****^, *p* < 0.0001.

Next, the downstream target proteins of BPGM‐mediated lactylation were screened and identified. The proteins immunoprecipitated with the lactylated protein antibody were subjected to LC‐MS/MS analysis. The procedures included protein extraction, peptide enzymatic digestion, mass spectrometry, and data analysis (Figure 7A). Figure 7B shows some of the top‐ranked proteins, which were upregulated in cells overexpressing *BPGM*. Among the differentially expressed proteins affected by *BPGM* in HCC cells, we focused on ret proto‐oncogene (RET), which might be an important downstream protein of BPGM according to its differential expression in HCC tissues (Figure 7C). Our co‐immunoprecipitation (Co‐IP) results validated that *BPGM* promotes the lactylation of RET (Figure 7D). In cells overexpressing *BPGM*, the expression level of RET was significantly increased (Figure 7E). The liver tissues from the *Bpgm*‐CKO mice showed decreased expression of RET protein (*p* < 0.05) (Figure 7F), which was consistent with the in vitro results. Since ubiquitination influences protein degradation, we hypothesized that *BPGM*‐mediated lactylation might compete with ubiquitination to prevent RET degradation. Using the DeepKla and GPS‐Uber websites to predict potential lactylation and ubiquitination sites on RET, we identified three candidate sites that were predicted for both modifications (Figure 7G). To identify the potential modification sites on RET, we evaluated the ubiquitination and lactylation levels obtained with mutations of each of the three sites (*RET*‐K523, *RET*‐K549, and *RET*‐K965). Firstly, we verified the overexpression efficiency of *RET*‐WT, *RET*‐K523R, *RET*‐K549R, and *RET*‐K965R (Figure 7H). The Co‐IP results showed that *RET*‐K549R eliminated both lactylation and ubiquitination of RET (*p* < 0.0001), while *RET*‐K523R and *RET*‐K965R mutations had no significant effect on the level of lactylation and ubiquitination (Figure 7I,J). Furthermore, treating PLC/PRF/5 cells with 2‐deoxy‐D‐glucose (2‐DG, a glucose inhibitor) to reduce lactate production showed a decrease in lactylation accompanied by an increase in ubiquitination (Figure 7K,L). P300 is a potential lactylation writer protein, and deprivation of P300 reduces histone lactylation [5, 9]. We used siRNA to knock down the expression of *P300* in PLC/PRF/5 cells (Figure 7M). The level of protein lactylation was decreased when *P300* was silenced (Figure 7N), and the ubiquitination of RET was significantly upregulated (Figure 7O). Together, these results indicated that BPGM could promote P300‐mediated lactylation and inhibit RET ubiquitination at the K549 site, thereby inhibiting its degradation, which ultimately leads to the upregulation of RET protein levels.

### 2.8 RET Mediated BPGM‐Induced Proliferation and Migration of HCC Cells

**FIGURE 8:**
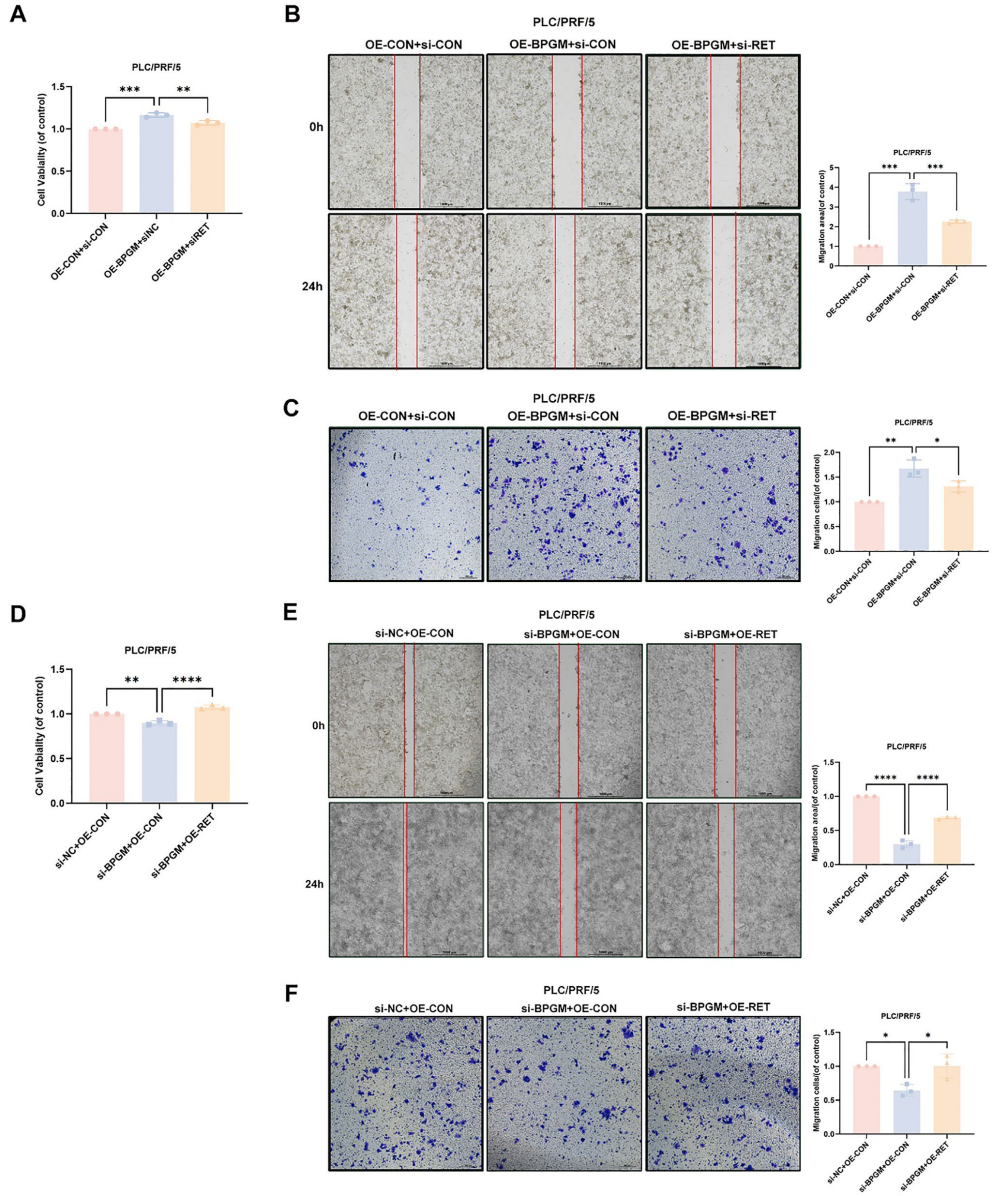
Roles of RET in BPGM‐mediated proliferation and migration capacity of HCC cells. (A–C) CCK‐8, wound‐healing, and Transwell assays of PLC/PRF/5 cells co‐transfected with *BPGM* plasmid DNA and *RET* siRNA (*n* = 3). (D‐F) CCK‐8, wound‐healing, and Transwell assays of PLC/PRF/5 cells co‐transfected with *BPGM* siRNA and *RET* plasmid DNA (*n* = 3). Data are presented as the means ± SDs. Statistical significance across groups was determined by one‐way ANOVA. ^*^, *p* < 0.05; ^**^, *p* < 0.01; ^***^, *p* < 0.001; ^****^, *p* < 0.0001.

Considering that BPGM could promote the expression level of RET protein, we investigated whether RET could mediate the function of *BPGM* during the progression of HCC. We silenced *RET* in PLC/PRF/5 cells overexpressing *BPGM*. Using CCK‐8, wound‐healing, and Transwell assays, we found that the knockdown of *RET* could significantly attenuate *BPGM*‐induced proliferation and migration ability of HCC cells (Figure 8A–C). And the *RET* overexpression could restore the decreased proliferation and migration of HCC cells mediated by *BPGM* knockdown(Figure 8D–F). The above results indicated that BPGM promotes the proliferation and malignant phenotype of HCC cells by promoting the expression of RET.

### 2.9 BPGM in HCC Cells Promotes M2 Polarization of Macrophages

**FIGURE 9:**
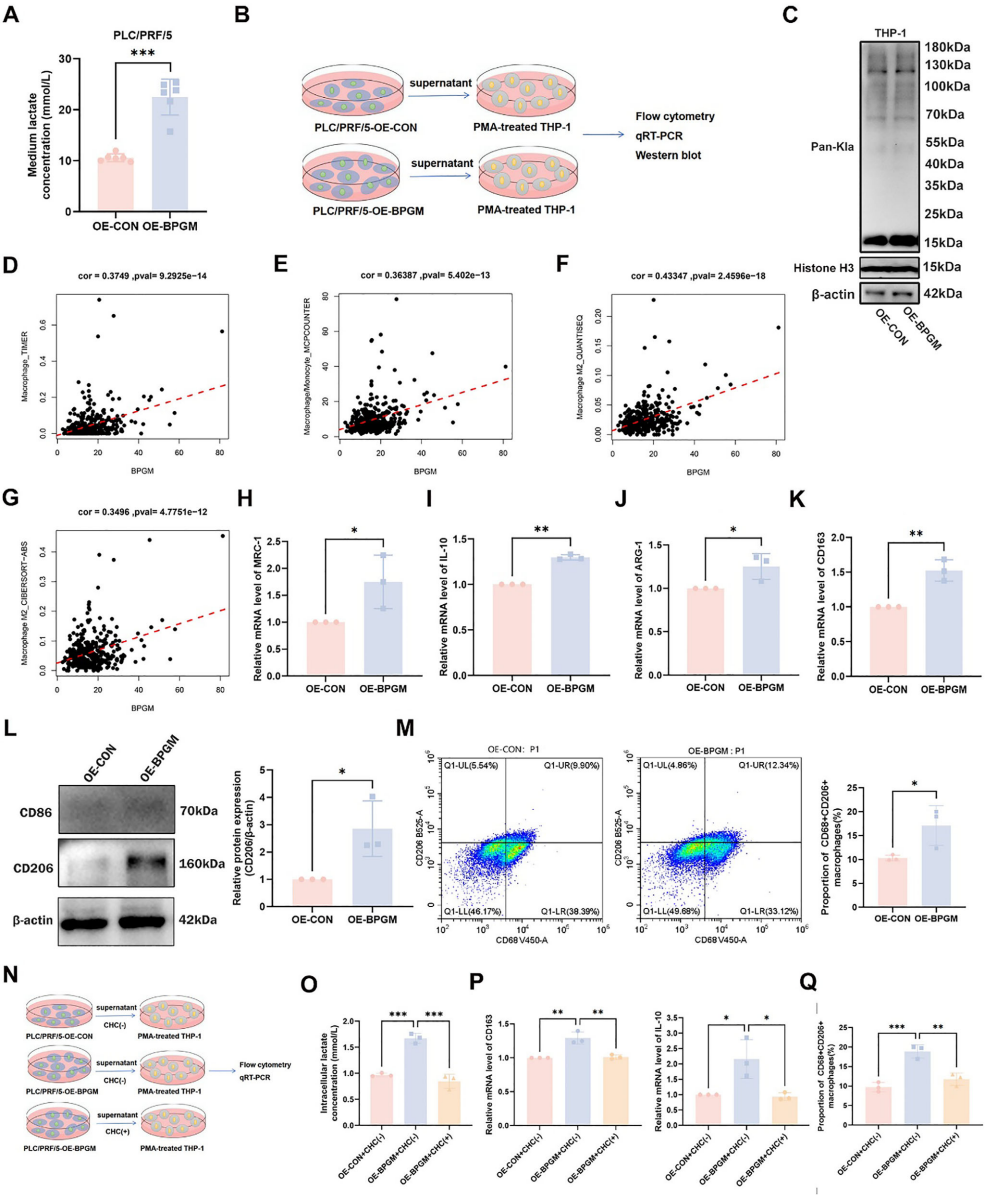
Overexpression of BPGM promotes microenvironmental M2 macrophage polarization. (A) Lactate concentration in culture supernatants of PLC/PRF/5 cells overexpressing *BPGM* for 72 h (*n* = 6). (B) Diagram of PLC/PRF/5 cell and PMA‐treated THP‐1 co‐culture model. (C) The levels of Pan Kla in the OE‐CON and OE‐*BPGM* groups were assessed using a Western blot. (D–G) Relationship between *BPGM* and immune cell infiltration based on the TIMER, MCPCOUNTER, QUANTISEQ, and CIBERSORT‐ABS algorithms. (H–K) qRT‐PCR analysis of *MRC‐1*, *IL‐10*, *ARG‐1* and *CD163* in the OE‐CON and OE‐*BPGM* groups (*n* = 3). (L) Western blot analysis of CD86 and CD206 protein levels in the OE‐CON and OE‐*BPGM* groups (*n* = 3). (M) The proportion of CD68+CD206+ macrophages between OE‐CON and OE‐*BPGM* groups was assessed by flow cytometry (*n* = 3). (N) Diagram of the co‐culture system established between PLC/PRF/5 cells and THP‐1 cells (pre‐treated with PMA, then exposed to CHC). (O) Comparison of intracellular lactate concentration across three experimental groups (*n* = 3). (P) qRT‐PCR analysis of *CD163* and *IL‐10* in the groups (*n* = 3). (Q) The proportion of CD68+CD206+ macrophages among the three groups was assessed by flow cytometry (*n* = 3). Statistical analyses were performed using a two‐tailed Student's *t*‐test between two groups. Statistical significance across three groups was determined by one‐way ANOVA. The data are presented as the means ± SDs. ^*^, *p* < 0.05; ^**^, *p* < 0.01.

We further verified the correlation between BPGM‐mediated HCC progression phenotype and macrophages in vitro. Compared with the PLC‐OE‐CON group, lactate concentration in the supernatant of PLC/PRF/5 cells was significantly increased after *BPGM* overexpression (PLC‐OE‐*BPGM*) (*p* < 0.001) (Figure 9A). Based on this phenomenon, we collected supernatants from PLC/PRF/5 cells with and without *BPGM* overexpression, and then used these supernatants to treat M0 macrophages differentiated from THP‐1 cells stimulated with phorbol 12‐myristate 13‐acetate (PMA) (Figure 9B). After co‐culture, western blot results showed that the protein lactylation level at approximately 15 kDa in macrophages was significantly upregulated in the OE‐*BPGM* group (Figure 9C), suggesting that lactate might promote M2 macrophage polarization through histone lactylation. TIMER and MCPCOUNTER algorithms were used to analyze the correlation between the expression level of *BPGM* in HCC tissues and macrophages in the microenvironment from the TCGA cohorts (Figure 9D,E). Assays using CIBERSORT‐ABS and QUANTISEQ algorithms also showed that the expression level of *BPGM* in HCC tissues was positively correlated with M2 macrophage infiltration (Figure 9F,G). Compared with the OE‐CON group, the mRNA expression levels of M2 macrophage markers (*MRC‐1*, *IL‐10*, *ARG‐1*, and *CD163*) were significantly upregulated in M0 macrophages differentiated from THP‐1 cells incubated with the supernatant of OE‐*BPGM* HCC cells (*p* < 0.01 or *p* < 0.05) (Figure 9H–K). The protein expression level of CD206 was significantly upregulated (*p* < 0.01) (Figure 9L). Flow cytometry results further confirmed that the proportion of CD68+CD206+ M2 macrophages was significantly higher in the OE‐*BPGM* group than in the OE‐CON group (*p* < 0.05) (Figure 9M). Additionally, M0 macrophages differentiated from THP‐1 cells were incubated with both the MCT‐specific inhibitor α‐cyano‐4‐hydroxycinnamic acid (CHC) and the aforementioned supernatants from PLC‐OE‐*BPGM* cells. A schematic of the co‐culture model is provided in Figure 9N. CHC reduced intracellular lactate accumulation, M2 marker (CD163, IL‐10) mRNA levels, and the proportion of CD68+CD206+ M2 macrophages in M0 macrophages induced by the supernatant from *BPGM*‐overexpressing HCC cells (Figure 9O–Q). All results show that CHC‐mediated inhibition of lactate transport significantly abrogates the M2 polarization‐promoting effects induced by the supernatant from *BPGM*‐overexpressing PLC/PRF/5 cells.

## 3 Discussion

At present, HCC remains associated with high mortality rates. And despite recent therapeutic advances, the survival rate of patients with advanced HCC is still low. Therefore, specific molecular targeted therapies have emerged as a key focus in HCC research. It has been reported that the expression level of *BPGM* is significantly upregulated during the progression from chronic hepatitis B (CHB) to HCC [17]. In this study, the significant upregulation of *BPGM* in HCC tissues was verified using the TCGA database and clinical samples of liver cancer. At the same time, we found that *BPGM* also showed good performance in the diagnosis and prognosis of HCC. Therefore, it may become a potential clinical marker for HCC. In this study, a series of functional experiments was performed to confirm that BPGM promotes HCC development by regulating the proliferation and migration of HCC cells.

The emergence of lactylation has led to a new understanding of lactate's function. Some studies have found that histone lactylation plays an important role in the transcriptional regulation of downstream target genes. For example, lactylation of histone H3K18 promotes the progression of idiopathic pulmonary fibrosis through the YTHDF1/m6A/NREP pathway [24]. Lactylation of histone H4K12 promotes the positive feedback of glucose metabolism in microglia, thereby mediating the progression of Alzheimer's disease [25]. TLR signaling receptor (B‐cell Adapter for PI3K, BCAP) induces the transformation of inflammatory macrophages into reparative macrophages through the modification of macrophages with lactate [26]. Our study used LC‐MS and found that the major proteins modified by intracellular lactate in HCC were non‐histone proteins with a focus on RET. RET is a proto‐oncogene that encodes a tyrosine kinase receptor that is activated by a ligand/co‐receptor complex, glial cell line‐derived neurotrophic factor (GDNF), and its ligand GFRα1. This leads to RET dimerization and phosphorylation. The activated RET then triggers downstream signaling through the RAS/mitogen‐activated protein kinase (MAPK) pathway and the phosphatidylinositol 3‐kinase (PI3K)/protein kinase B (AKT) pathway [27, 28]. It has been reported that the expression of RET protein is significantly upregulated in hepatocellular carcinoma tissues compared with adjacent tissues. This was determined by IHC analysis of HCC and adjacent tissues [29]. In this study, we revealed that BPGM‐mediated RET expression could promote the proliferation and migration of HCC cells, and identified lactylation of the *RET*‐K549 site.

Macrophages are one of the key mediators within the TME of cancers, including HCC [30]. A high proportion of macrophages in tumor tissues is usually correlated with a poor prognosis [31, 32]. Macrophages in tumors are predominantly classified into two groups: the M1 inflammatory phenotype and the M2 pro‐tumor phenotype [33]. Changes from M2 macrophages to M1 macrophages inhibit tumor growth and could treat solid tumors [34]. Lactate is reported to induce the polarization of macrophages [35]. In this study, the overexpression of BPGM in HCC cells promoted protein lactylation and induced M2 polarization of macrophages, which promoted the progression of HCC.

There are still some limitations in our study that need to be acknowledged. Firstly, our in vivo findings were primarily derived from the DEN‐induced HCC model. The validation in additional HCC models with diverse etiologies, along with data from well‐annotated clinical cohorts stratified by etiology, will help enhance the generalizability of our conclusions. Secondly, direct in vivo evidence for *RET*‐K549 lactylation remains lacking due to current technical constraints, and future work will establish a *RET*‐K549 mutation knock‐in mouse model to verify the physiological relevance of this site‐specific modification in BPGM‐mediated tumor progression.

## 4 Conclusion

**FIGURE 10:**
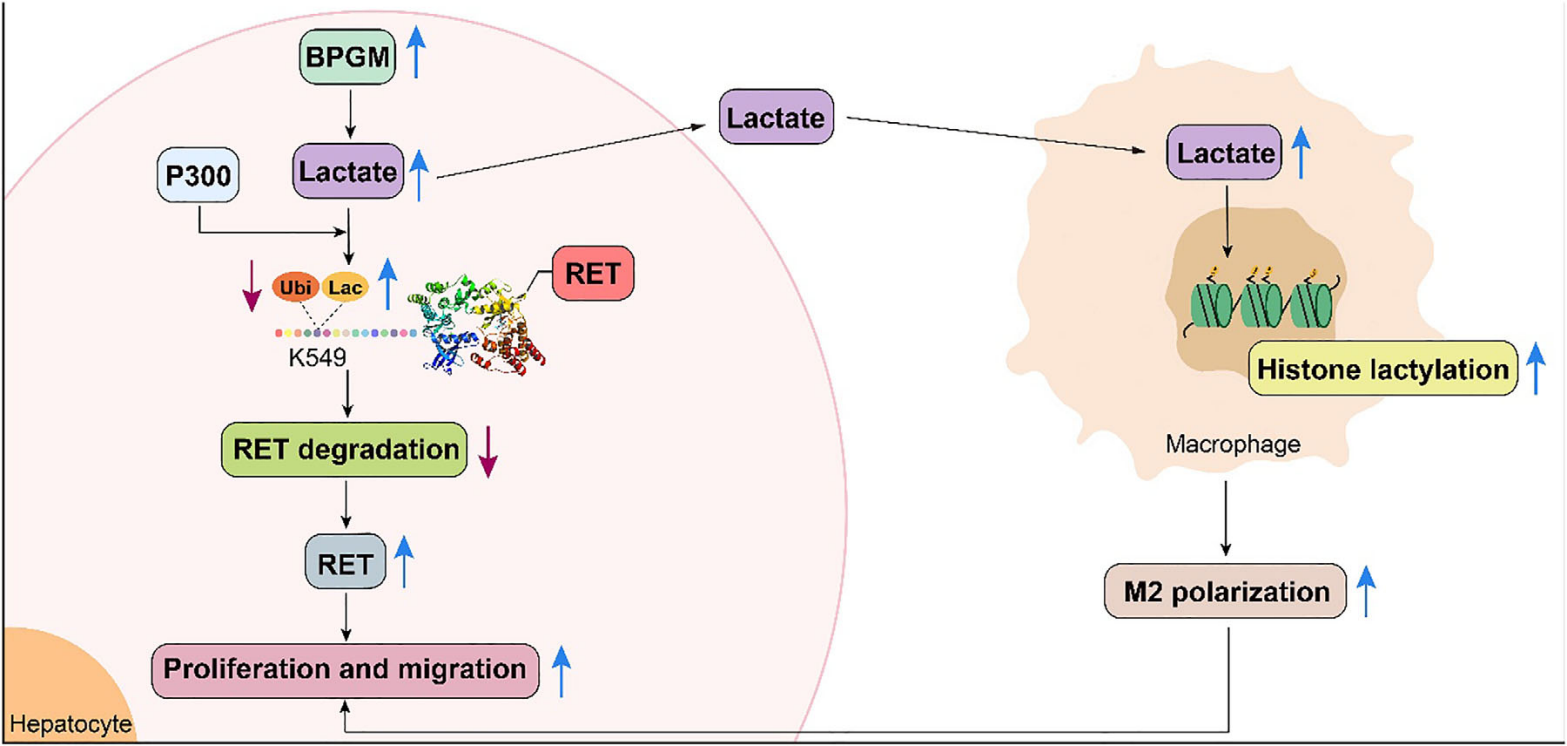
Mechanistic schematic diagram.BPGM promotes the expression level of RET by increasing the lactylation of *RET*‐K549 and inhibiting its ubiquitination levels in HCC cells. Furthermore, BPGM in HCC cells could also promote M2 polarization in macrophages through lactate secretion. Both of the mechanisms could promote the progression of HCC.

In summary, this study reveals a novel role for BPGM in promoting HCC tumorigenesis. BPGM induced lactylation of RET protein at K549, competitively inhibiting ubiquitination at the same residue. This increased the expression level of RET, mediating BPGM's promotion of HCC cell proliferation and migration. In addition, BPGM in HCC cells promoted lactate accumulation in the TME, increased histone lactylation levels in macrophages, and induced M2 polarization of macrophages, which could also contribute to the tumorigenesis of HCC (Figure 10). These data offer new insights into the molecular mechanisms of HCC occurrence and suggest that BPGM could be a promising target for HCC treatment.

## 5 Experimental Section

### 5.1 Generation of Hepatocyte‐Specific *Bpgm*‐Knockout Mice and Hepatocarcinogenesis Induction

The *Bpgm*‐flox heterozygous (*Bpgm* ^+/−^) mice required for this study were purchased from GemPharmatech Co., Ltd (Jiangsu, China). Hepatocyte‐specific *Bpgm*‐knockout mice were generated by crossing *Bpgm* ^fl/fl^ mice with *Alb‐Cre* transgenic mice. Genotyping confirmed the experimental group (*Bpgm*‐CKO, *Bpgm* ^CKO^, *Cre*‐positive) and control group (*Bpgm*‐CON, *Bpgm* ^fl/fl^, *Cre*‐negative). To induce HCC, 15‐day‐old male mice received a single intraperitoneal injection of diethylnitrosamine (DEN; 25 mg/kg body weight). The mice were subsequently maintained on a standard chow diet and water ad libitum until sacrifice at 52 weeks of age. All animal procedures were conducted in compliance with the institutional animal care guidelines approved by the animal ethics committee of the Eighth Affiliated Hospital of Sun Yat‐sen University (approval number: 2023‐051‐01). The following primers were used for PCR analysis in this study (Table S1).

### 5.2 RNA Extraction, Reverse Transcription, and PCR

Extraction of total RNA was performed using TRIzol reagent (Takara, Japan) according to the manufacturer's recommended procedure. Single‐strand cDNA was generated with the Evo M‐MLV reverse transcription kit (AG, China). Quantitative real‐time PCR (qRT‐PCR) was performed using SYBR Green Premix Pro Taq HS qPCR kit (AG, China). The following primers were used for qRT‐PCR analysis in this study (Table S2).

### 5.3 Western Blot

Cell and tissue lysates were prepared using RIPA lysis buffer (Beyotime, China), followed by centrifugation at 12 000 × *g* for 20 min at 4°C. Protein samples were separated using 7.5% or 10% SDS‐PAGE and subsequently transferred onto polyvinylidene fluoride membranes (Millipore Corporation, USA). After blocking with 5% skim milk powder for 1.5 h at room temperature, the membranes were incubated at 4°C overnight with the primary antibodies. The membranes were then incubated with enzyme‐conjugated secondary antibodies (Proteintech, China). The band signals were visualized using chemiluminescence imaging (e‐BLOT, China). The primary antibodies used in this study are listed in Table S3.

### 5.4 Hematoxylin‐Eosin Staining

To examine the pathological alterations in liver tissues across different experimental groups, the harvested livers were fixed in 4% paraformaldehyde. Using standard histological techniques, 3‐µm‐thick sections were prepared from the embedded paraffin blocks. Following deparaffinization and rehydration, the sections were stained using a Hematoxylin and Eosin (H&E) staining kit for histological evaluation (Servicebio, China).

### 5.5 Immunohistochemical Staining

After dewaxing and rehydration, antigen retrieval was performed using an appropriate buffer. Endogenous peroxidase activity was blocked with 3% H_2_O_2_. Sections were incubated with 3% BSA for blocking, followed by overnight incubation at 4°C with the primary antibody (BPGM, 1:200, Proteintech; Ki‐67, 1:500, servicebio; alpha‐smooth muscle actin (α‐SMA), 1:500, servicebio). After PBS washes, an HRP‐conjugated secondary antibody was applied and incubated for 50 min. DAB was used for color development, and hematoxylin was applied for counterstaining. Finally, the sections were dehydrated, cleared, and mounted for microscopic examination. The staining intensity was graded on a scale of 0 to 3: 0 (negative), 1 (weakly positive), 2 (moderately positive), and 3 (strongly positive). The H‐score was calculated using the formula: (3 × percentage of strongly positive cells) + (2 × percentage of moderately positive cells) + (1 × percentage of weakly positive cells). The primary antibodies used in this study are listed in Table S3.

### 5.6 Sirius Red Staining

Sirius red staining was performed to evaluate the extent of fibrosis in the tumor tissues. Briefly, paraffin‐embedded tumor sections were dewaxed and incubated with Sirius red solution (Solarbio, China) for 1 h. After washing under running water for 10 min, the sections were dehydrated through a graded ethanol series and mounted with neutral gum. Randomly selected fields from each section were examined under an inverted microscope (Olympus) to assess the staining results.

### 5.7 Multiplex Immunofluorescence Staining

First, paraffin sections of mouse liver tissue were subjected to dewaxing. Antigen retrieval was then performed. After gently blotting the sections dry, a hydrophobic barrier pen was used to circle the tissue, followed by the application of 3% BSA for 30 min for blocking. Primary antibodies are these: CD68 (Servicebio, China), CD206 (Servicebio, China), and CD86 (Servicebio, China), and the sections were incubated overnight at 4°C in a humidified chamber. Subsequently, the appropriate secondary antibody was applied and incubated for 50 min at room temperature in the dark. For nuclear counterstaining, the sections were incubated with DAPI for 10 min at room temperature, protected from light. Finally, the sections were mounted using an anti‐fade mounting medium and imaged under a fluorescence microscope. The primary antibodies used in this study are listed in Table S3.

### 5.8 Spatial Transcriptomics Analysis

Raw data generated by the Stereo‐seq platform were processed using the official Stereo‐seq Analysis Workflow (SAW) for alignment and gene expression matrix generation. The resulting data were loaded into the Seurat R package (v4.3.0) for downstream analysis. Bins were filtered based on quality control criteria, excluding those with fewer than 200 detected genes or a mitochondrial gene percentage exceeding 20%. Gene expression was subsequently normalized using the “SCTransform” function.

To assign cell type identities to spatial bins, a reference‐based deconvolution strategy was employed. First, the single‐cell RNA‐seq dataset was comprehensively annotated through a combination of automated annotation with the SingleR package and manual validation against canonical marker genes identified from the literature. This annotated dataset then served as a reference to deconvolve the spatial data. Cell type identities were mapped onto each spatial bin using Seurat's anchor‐based integration workflow, specifically with the “TransferData” function. Following the annotation, further spatial analyses were conducted. To quantify local cellular interactions, the average proportion of various cell types within a 40 µm radius of hepatocytes was calculated. Distinct tissue microenvironments (niches) were identified by applying unsupervised k‐means clustering (k = 5) to the spatial coordinates and gene expression profiles.

To investigate the relationship between hepatocyte density and immune infiltration, a module score for hepatocyte markers was calculated for each bin using “AddModuleScore”. Bins were then ranked by this score and stratified into deciles to compare the proportion of monocyte/macrophage cells between groups in high‐density regions. Statistical significance for niche and density‐based comparisons was assessed using a Wilcoxon rank‐sum test.

### 5.9 Single Cell Sequencing Analysis

Raw sequencing data were processed using Cell Ranger (v6.1.0) against the s+ to generate feature‐barcode matrices. Subsequent analysis was conducted in the Seurat R package (v4.3.0). After removing low‐quality cells (gene counts outside the 500–6,000 range; mitochondrial DNA > 20%), the data were normalized using “SCTransform”, and inter‐sample batch effects were corrected using the Harmony algorithm. Following principal component analysis, the top 30 principal components were used for UMAP dimensionality reduction and graph‐based clustering (“FindClusters”, resolutio*n* = 0.8), yielding 39 distinct clusters. These clusters were manually annotated into 18 major cell types based on the expression of canonical marker genes identified through differential expression analysis (“FindAllMarkers”) and literature reviews. To assess the activity of proliferation pathways, the expression of Myc, Met, Jun, Ccnd1, Fos, and Axin2 was compared between the experimental groups within the malignant hepatocyte population using a Wilcoxon rank‐sum test. For in‐depth characterization of macrophages, this population was subsetted and re‐clustered, identifying 11 subsets. These clusters were then classified as M2‐polarized tumor‐associated macrophages (M2 TAMs) or non‐M2 TAMs based on the expression of *Mrc1* (M2 marker) and *Il1b* (M1 marker). The percentage of M2 TAMs was calculated for each sample, and the statistical significance of the difference between groups was determined using a Wilcoxon rank‐sum test (*p* < 0.05).

### 5.10 Cell Lines and Cell Cultures

The Huh7 (RRID: CVCL_0336) and PLC/PRF/5 (RRID: CVCL_0485) cell lines were purchased from Shanghai Fusheng Biotechnology, which also performed STR profiling and mycoplasma contamination testing. The THP‐1 (RRID: CVCL_0006) cell line was purchased from Shanghai Genechem. STR profiling and mycoplasma contamination testing of the cells were conducted by Shanghai Genechem. For cell culture, the Huh7 and PLC/PRF/5 cells were grown in DMEM (Gibco, USA). The THP‐1 cells were cultured in RPMI 1640 medium (Gibco, USA). The cells were maintained at 37°C under a humidified 5% CO_2_ atmosphere in medium containing 10% certified heat‐inactivated fetal bovine serum (FBS, BI, Israel) and penicillin‐streptomycin (Biosharp, China).

### 5.11 siRNAs and Transfection

siRNAs targeting *BPGM, RET*, and *EP300* were commercially procured (GenePharma, China). siRNAs were transfected into cells using HiperFect Transfection Reagent (QIAGEN, Germany) according to the manufacturer's instructions. A list of siRNAs used appears in Table S4.

### 5.12 Cell Proliferation Assays

A total of 3000 cells were seeded into 96‐well plates (Nest, China) with 100 µL of complete medium. Cell proliferation was analyzed using the CCK‐8 assay (Vazyme, China). The CCK‐8 reagent was added to each well at the indicated time points, and after 2–3 h of incubation at 37°C, the absorbance was detected at a wavelength of 450 nm. The difference in cell viability between different groups was compared.

### 5.13 Colony Formation Assay

A total of 2000 cells suspended in 2.5 mL of complete medium were seeded into each well of a 6‐well plate, with medium replacement every 2–3 days. After 10–14 days of culture, colonies were fixed and stained with 0.1% crystal violet (Beyotime, China).

### 5.14 Transwell Assay

A 24‐well Transwell system (Corning, USA) with polycarbonate filters was used in the Transwell assay. A total of 1.0 × 105 cells suspended in serum‐free medium were placed in the upper chamber, and 700 µL of complete medium with 20% FBS was added to the lower chamber. Following a 24‐h or 48‐h incubation, cells in the Transwell system were fixed and stained with 0.1% crystal violet. The migrated cells on the lower membrane surface were then visualized and quantified.

### 5.15 Lactate Concentration Measurement

The lactate levels were measured using a Lactic Acid assay kit (Jiancheng, Nanjing) according to the manufacturer's instructions. The supernatants of lysed tumor cells or the supernatants of cell cultures were collected by centrifugation at 2500 rpm for 10 min. Distilled water (20 µL) was added to the blank tube, and the standard substance (20 µL, with a concentration of 3 mmol/L) was added to the standard tube for testing. Then, 20 µL of supernatant was added to the test tube. The enzyme working solution was then added to each tube. After mixing well, the chromogenic solution (200 µL) was added and incubated for 10 min at 37°C. Finally, the stop solution (2 mL) was added, the absorbance was measured at a wavelength of 530 nm, and the lactate concentration was calculated.

### 5.16 Co‐Immunoprecipitation

Total protein was extracted from the cells using NP‐40 lysis buffer (MedChemExpress, USA). The protein extracts were divided into three groups: Input, IgG, and IP. The IgG and immunoprecipitation (IP) groups were incubated with IgG (Proteintech, China) or the respective primary antibody overnight at 4°C. Subsequently, these mixtures were incubated at 4°C with Protein A/G Plus‐Agarose (Santa Cruz, USA) for 5 h. The agarose beads were then collected and washed three times with NP‐40 washing buffer. After elution, the beads were boiled in loading buffer, and the samples were resolved by SDS‐PAGE, followed by immunoblotting analysis.

### 5.17 Co‐Culture of OE‐BPGM PLC/PRF/5 Cells with THP‐1‐Derived Macrophages

First, BPGM was overexpressed in PLC/PRF/5 cells. Conditioned medium from BPGM‐overexpressed PLC/PRF/5 cells (OE‐BPGM PLC/PRF/5) was collected after 72 h of overexpression and then added to THP‐1 cells that had been differentiated into M0 macrophages by treatment with 100 ng/mL phorbol 12‐myristate 13‐acetate (PMA, MedChemExpress, USA) for 24 h. Following 24 h of co‐culture, the cells were harvested. Alpha‐cyano‐4‐hydroxycinnamate (CHC, MedChemExpress, USA) was dissolved in dimethyl sulfoxide (DMSO; Solarbio, China) to 7.5 mm.

### 5.18 Flow Cytometry

To generate a single cell suspension, harvested cells were washed twice with PBS. Intracellular staining was performed using an Intracellular Fixation/Permeabilization Buffer Kit (E‐CK‐A109, Elabscience, China). The single cell suspension was then incubated with Human TruStain FcX (Fc Receptor Blocking Solution, 422301, Biolegend, USA) at room temperature for 10 min, followed by incubation with intracellular antibodies for 30 min at room temperature. After washing, the cells were resuspended in Cell Staining Buffer (E‐CK‐A107, Elabscience, China). Flow cytometric analysis was performed using a Beckman Coulter CytoFLEX instrument (Beckman, USA). Data were collected, and subsequent analysis was conducted using CytExpert software. All fluorophore‐conjugated antibodies are listed in Table S5.

### 5.19 TCGA and GEO Datasets

RNA‐sequencing data and clinical profiles from 371 HCC patient samples, along with 50 matched adjacent normal tissues, were downloaded from The Cancer Genome Atlas (TCGA, https://portal.gdc.cancer.gov/) dataset. The gene expression GEO datasets (https://www.ncbi.nlm.nih.gov/geo/), including GSE14520 (*n* = 41) and GSE62232 (*n* = 91), were used to validate the expression level of BPGM. The HCC scRNA‐seq dataset GSE149614 (8 non‐tumor and 10 tumor samples) and GSE202642 (4 non‐tumor and 7 tumor samples) were also downloaded from the GEO database.

### 5.20 Patients and Specimens

We used 40 pairs of human HCC tissues and para‐cancerous tissues collected at the Eighth Affiliated Hospital of Sun Yat‐sen University. The Human Research Ethics Committee approved this study (approval number: 2025‐001‐01).

### 5.21 Statistical Analysis

Statistical analyses were performed using GraphPad Prism (version 9.5, GraphPad Software, San Diego, CA, USA). Data are presented as mean ± standard deviation (SD). The sample size (n) for each experiment is provided in the figure legends. The specific statistical tests used were as follows: For comparisons between two groups, a two‐tailed Student's *t*‐test was applied. For comparisons among three or more groups, one‐way analysis of variance (ANOVA) was performed. The diagnostic accuracy of biomarkers was compared using DeLong's test for receiver operating characteristic (ROC) curves. Survival analysis was conducted using the Kaplan‐Meier method, and differences between curves were assessed with the log‐rank test. Correlations between variables were evaluated using Spearman's rank correlation coefficient. *p*‐Value of less than 0.05 was considered statistically significant. In figures, significance is denoted as ^*^ *p* < 0.05, ^**^ *p* < 0.01, ^***^ *p* < 0.001, and ^****^ *p* < 0.0001; nonsignificant differences are indicated by “ns”.

## Author Contributions

M.Z., Y.Q.Z., L.K.Z., and L.S. acquired the funding; J.J.Z., L.S., L.L.L., Y.Z., and M.Z. performed the experiments; L.L.W., Y.X., and P.H. developed the methodology; J.J.Z., L.S., and L.K.Z. wrote the original draft and prepared all the figures; L.K.Z. and L.S. revised the manuscript. All authors reviewed the manuscript.

## Funding

This study was supported by the National Nature Science Foundation of China (Nos.82472353, 32170708, 82502816), Taishan Scholar Foundation of Shandong Province for LKZ (tsqn202312389), the Excellent Medical Innovation Talents Program of the Eighth Affiliated Hospital of Sun Yat‐sen University (Grant No. YXYXCXRC202402), and the Futian Healthcare Research Project (No. FTWS032), Zhejiang Provincial Natural Science Foundation of China (LBY22H200001).

## Conflicts of Interest

The authors declare no conflict of interest.

## Supporting information




**Supporting File**: advs73732‐sup‐0001‐SuppMat.docx.

## Data Availability

The data that support the findings of this study are available from the corresponding author upon reasonable request.
