# Supplementary material for: Hepatocyte BPGM Induces RET Lactylation and Macrophage Reprogramming to Promote Tumorigenesis in Hepatocellular Carcinoma
Source: Adv Sci (Weinh). 2026 Jan 9;13(16):e18180. doi: 10.1002/advs.202518180 (PMC13042787; doi:10.1002/advs.202518180)
Supplement: Supplementary file 1 — Supporting File: advs73732‐sup‐0001‐SuppMat.docx. [file ADVS-13-e18180-s001.docx]

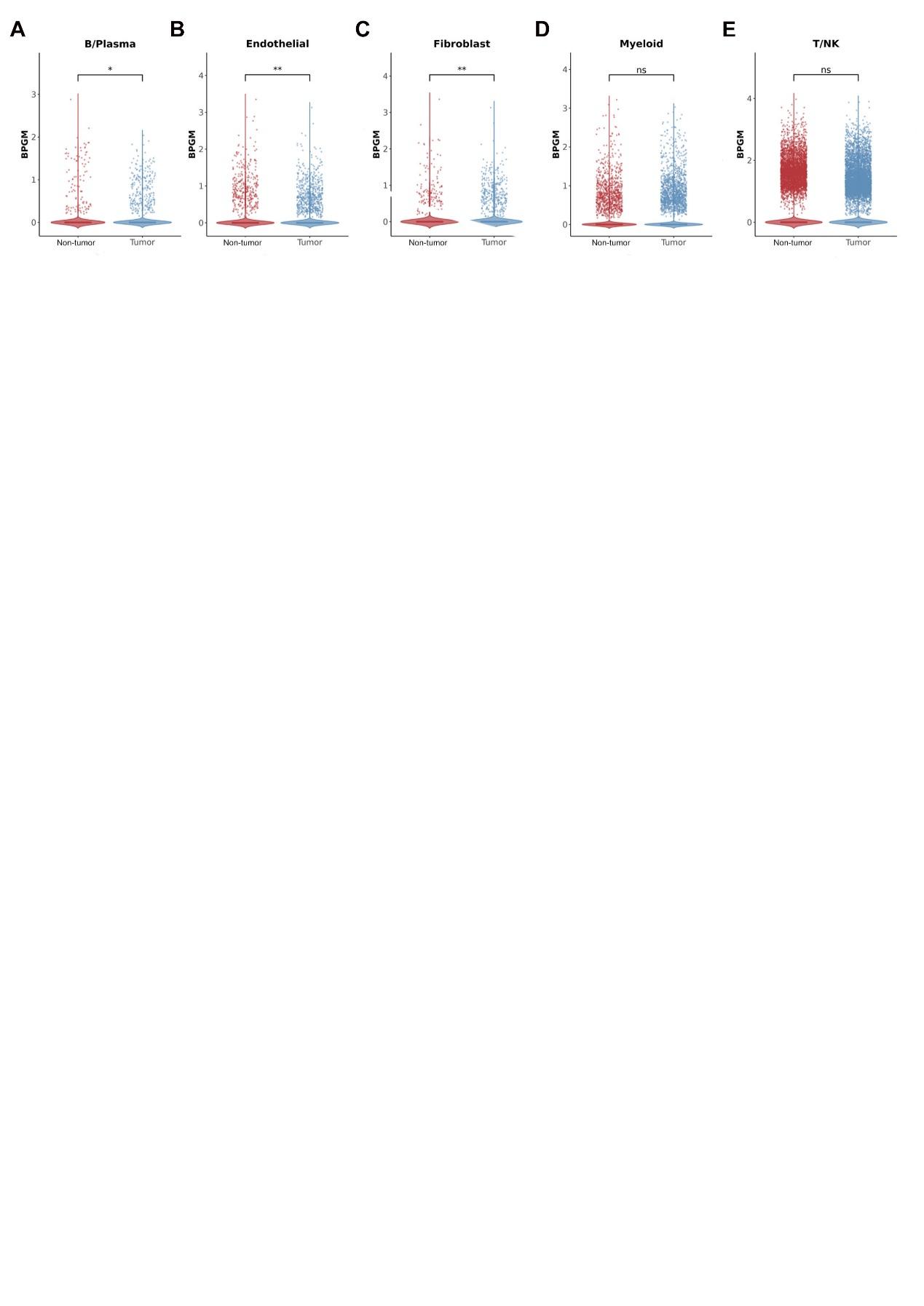


**Supplementary Figure 1.** Single cell RNA sequencing analysis of GSE149614 combined with GSE202642 dataset. (A-E) *BPGM* expression in B/Plasma cells, endothelial cells, fibroblast, myeloid cells and T/NK cells. The data are presented as the means ± SDs. *P* values were calculated using two-tailed Student’s t-test. ns, no significance; *, *P* < 0.05; **, *P* < 0.01.


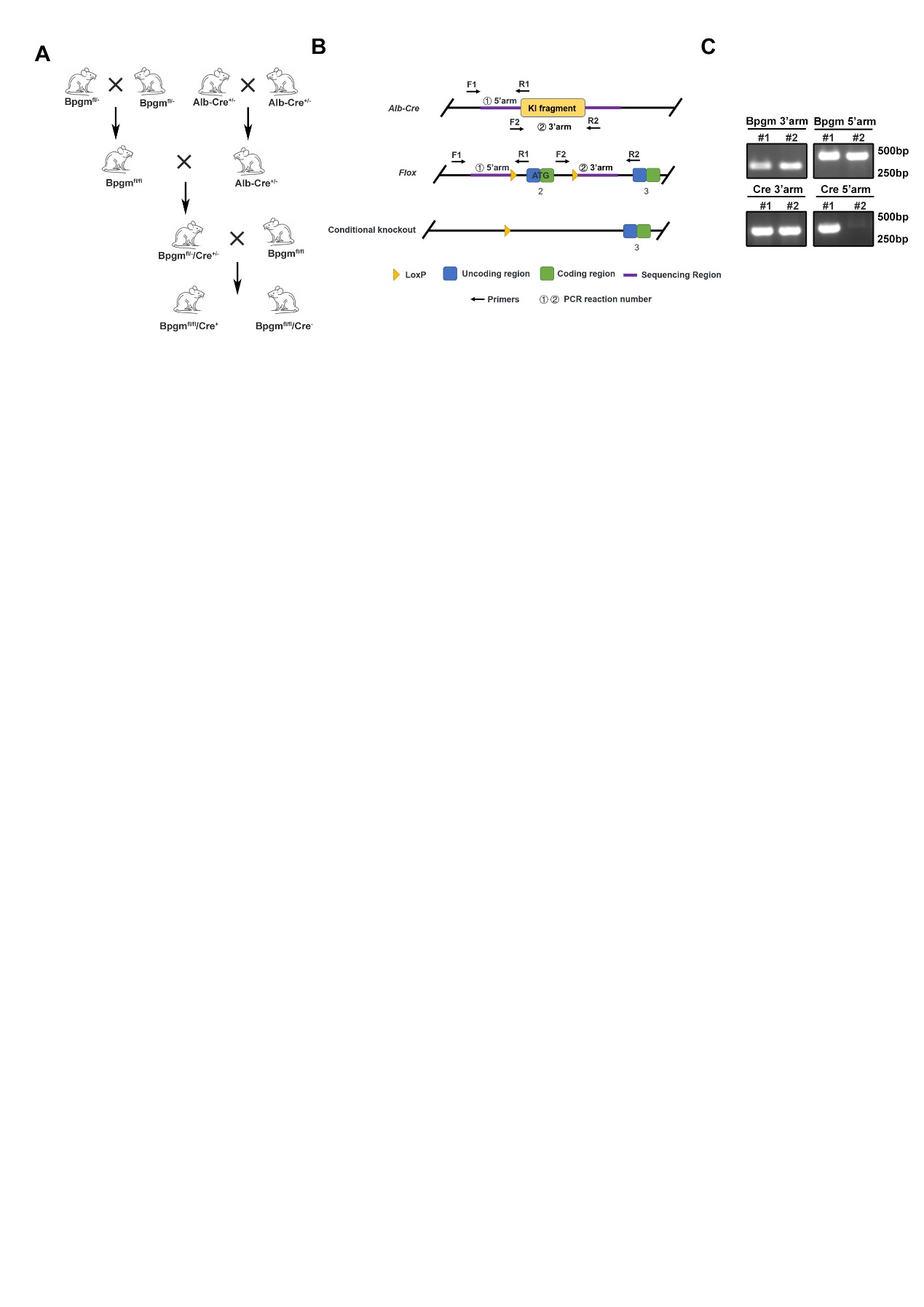


**Supplementary Figure 2.** Establishment of hepatocyte-specific *Bpgm* knockout mice. (A) The mating strategy for the *Bpgm*-knockout mice. (B) Strategy of genetyping. *Alb-Cre*: Genotypes were determined by PCR analysis: wild-type samples yielded only the wild-type amplicon; heterozygotes yielded both the wild-type and targeted amplicons; and homozygotes yielded only the targeted amplicon. Flox: Wild type: ① single WT band of PCR; ② no band of PCR. Heterozygote: ① A WT band and a Targeted band of PCR; ② A Targeted band of PCR. Homozygote: ① A single Targeted band of PCR; ②A Targeted band of PCR. Conditional knockout mice were generated by crossing mice harboring the floxed allele (Flox/Flox) with *Alb-Cre* mice. (C) Genotyping of the hepatocyte-specific *Bpgm*-knockout mouse model. For example, mouse #1 was *Bpgm* homozygous and Cre heterozygous (knockout group), whereas mouse #2 was Bpgm homozygous and Cre-negative (non-knockout control group).


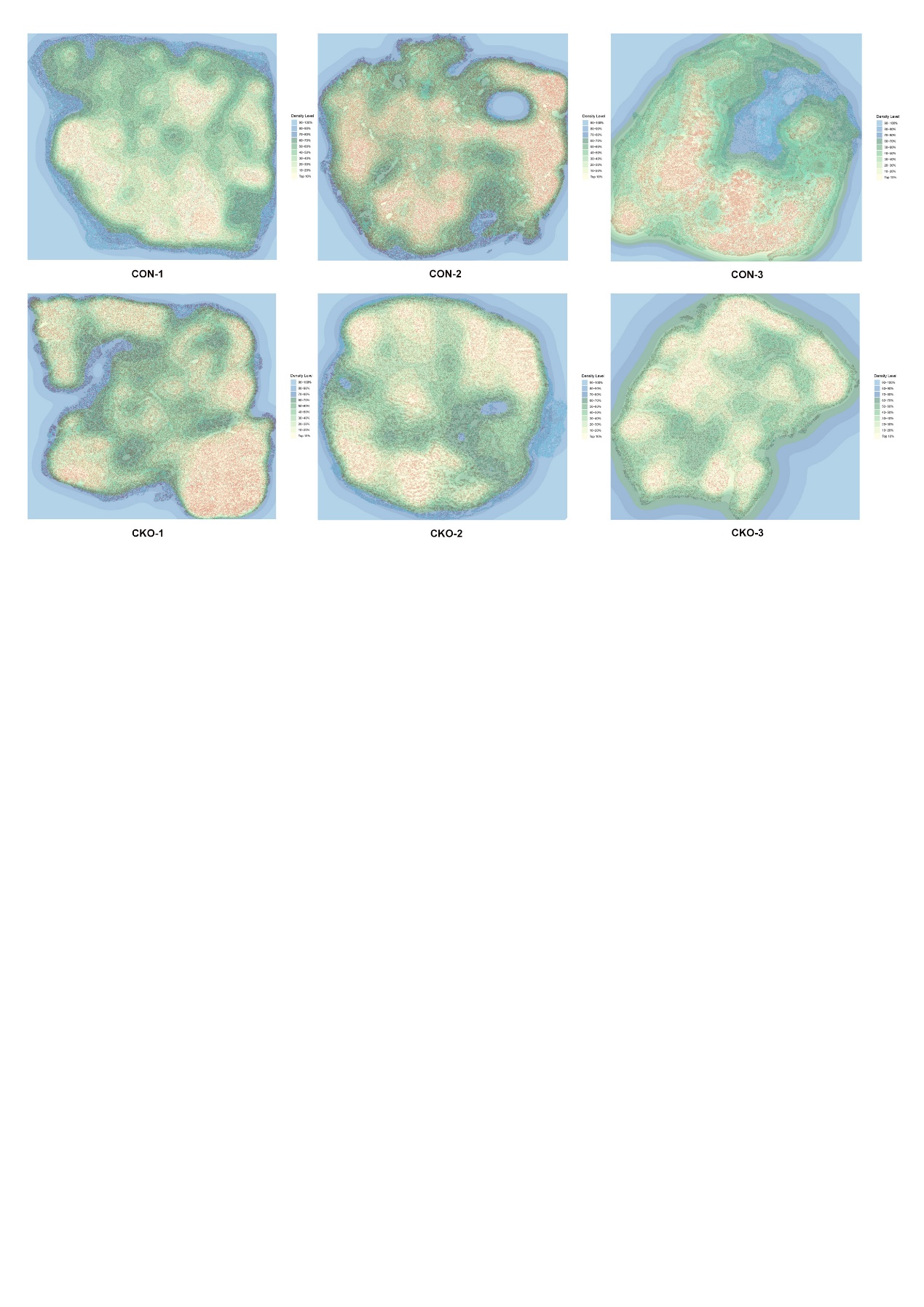


**Supplementary Figure 3.** Spatial Density Distribution of Hepatocytes. Division: Top 10%, 10%-20%, 20-30%, 30-40%, 40-50%, 50-60%, 60-70%, 70-80%, 80-90%, 90-100%.


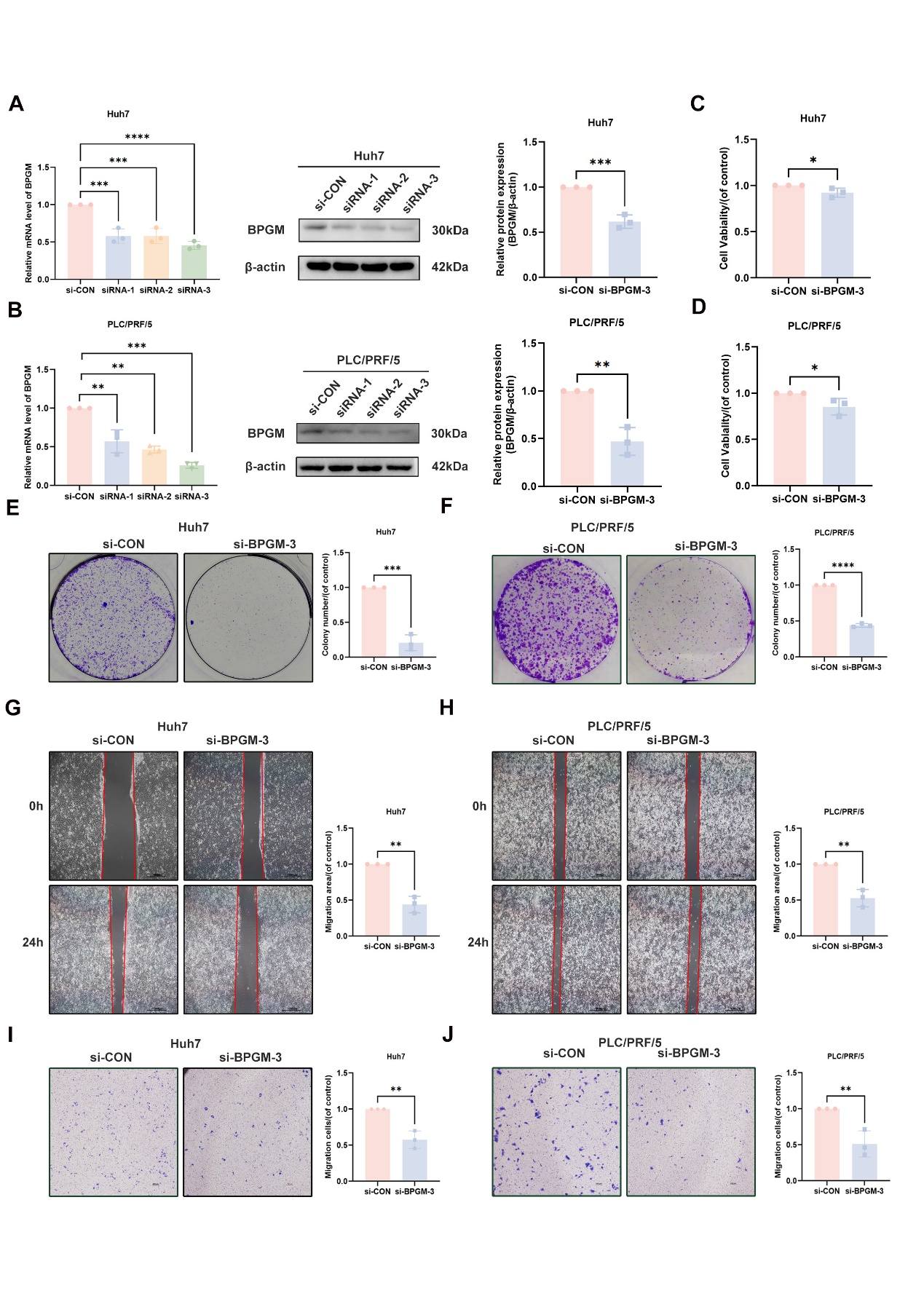


**Supplementary Figure 4.** Knockdown of *BPGM* reduces HCC cells proliferation and migration abilities (si-*BPGM*-3). (A, B) qRT-PCR and western blot analysis of BPGM expression level in *BPGM*-knockdown Huh7 and PLC/PRF/5 cells (n = 3). (C-J) CCK-8, colony formation, Wound-healing and Transwell assays in *BPGM*-knockdown Huh7 and PLC/PRF/5 cells (n = 3). Data are presented as the means ± SDs. *P* values were calculated using two-tailed Student’s t-test. *, *P* < 0.05; **, *P* < 0.01; ***, *P* < 0.001; ****, *P* < 0.0001.


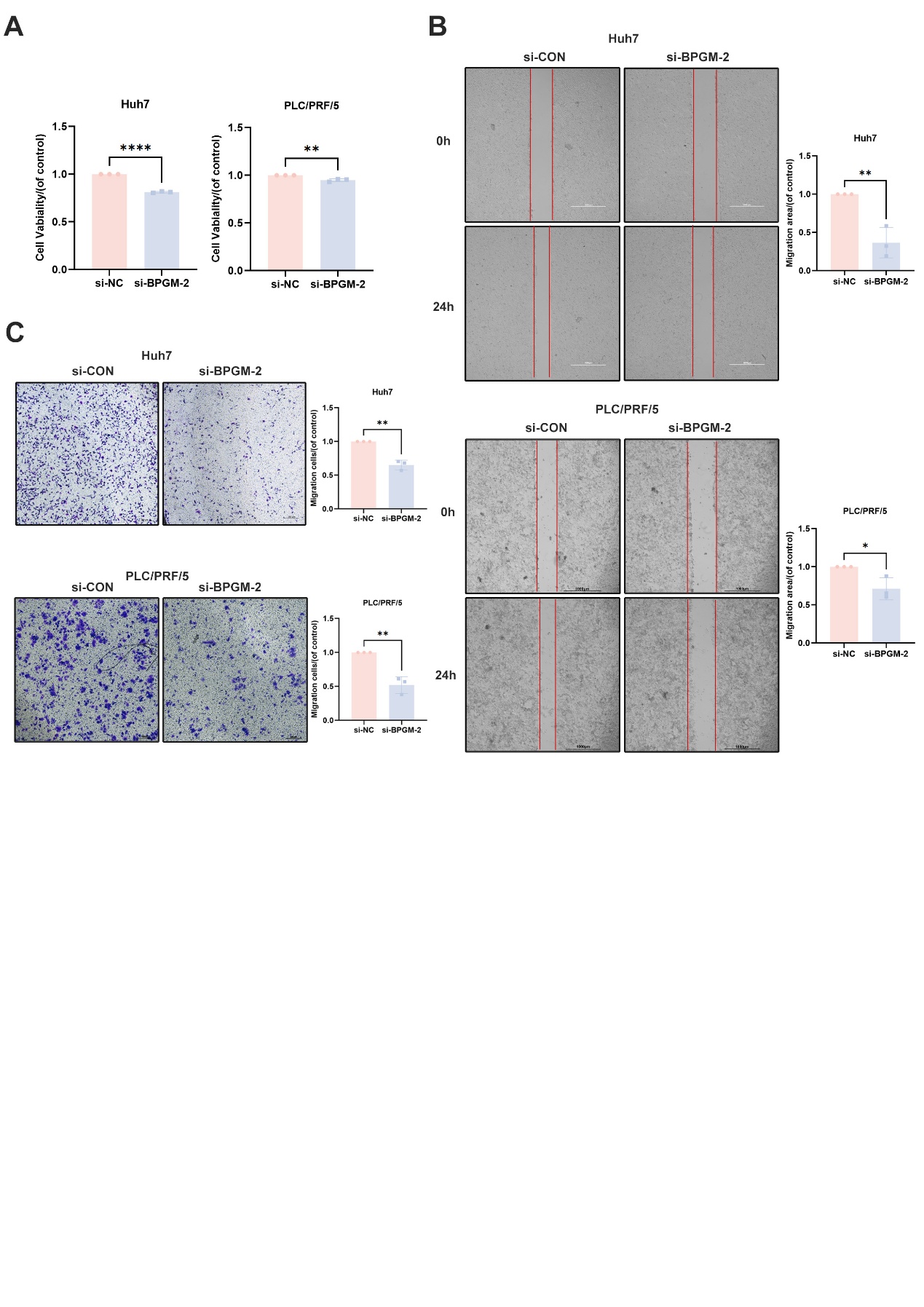


**Supplementary Figure 5.** Knockdown of *BPGM* reduced HCC cells proliferation and migration abilities (si-*BPGM*-2). (A-C) CCK-8 and Transwell assays were performed in BPGM-knockdown Huh7 and PLC/PRF/5 cells (n = 3). The data are presented as the means ± SDs. Two-tailed Student’s t-test was used to evaluate statistical significance. *, *P* < 0.05; **, *P* < 0.01; ****, *P* < 0.0001.


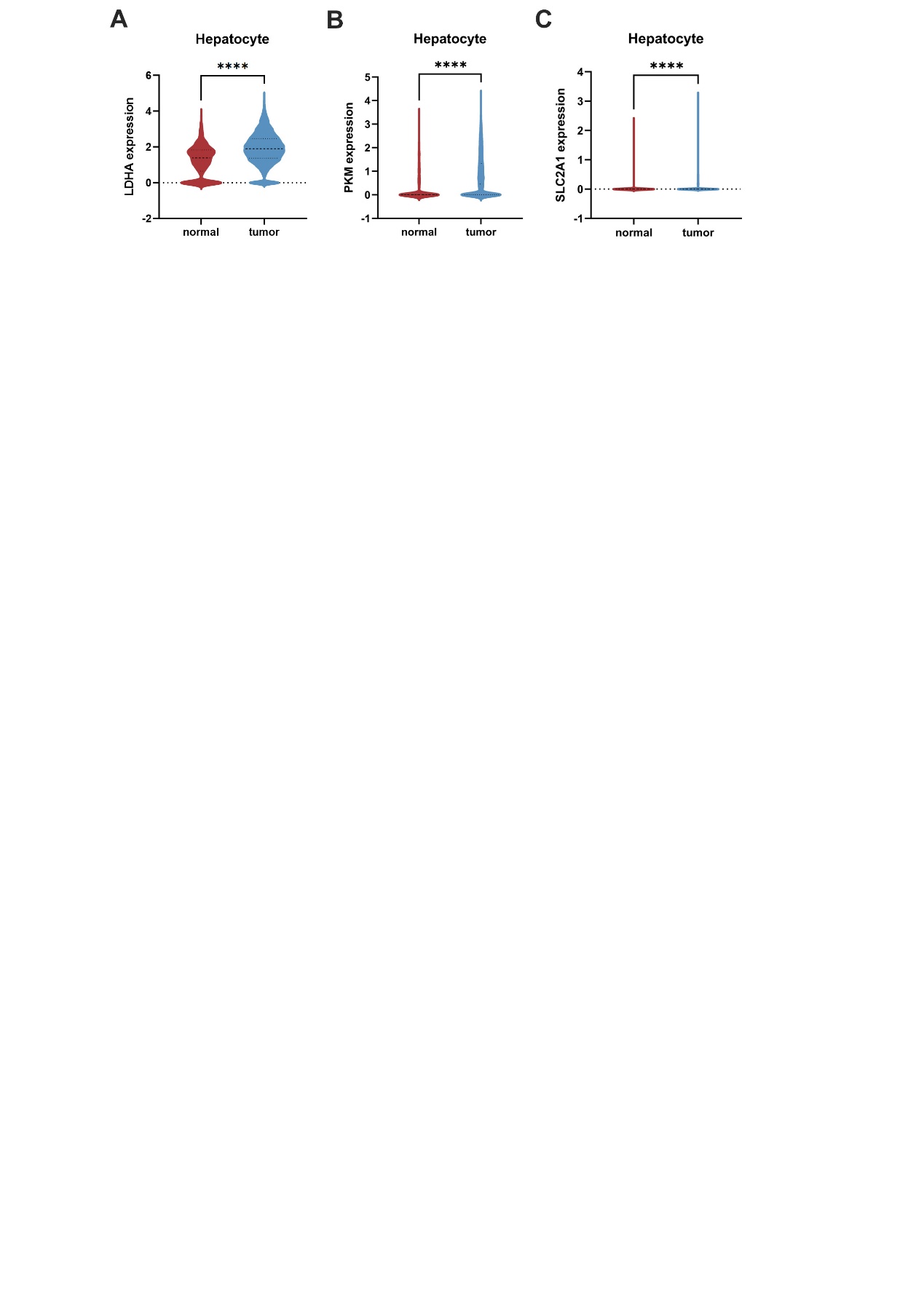


**Supplementary Figure 6.** BPGM promotes lactate production and lactylation. (A-C) Violin plot showed *LDHA, PKM and* *SLC2A1* expression level in hepatocytes from GSE149614 dataset. The data are presented as the means ± SDs. Two-tailed Student’s t test was used. ****, *P* < 0.0001.

Supplementary Table 1. Primer sequences for mouse genotyping

| **Primer (PCR)** | **Sequence (5’-3’)** |
| --- | --- |
| **Bpgm 3′-Forward** | 5′-TCTGAGGCGGAAAGAACCAG-3′ |
| **Bpgm 3′-Reverse** | 5′-CTCAGATGCCAAGAGACAGATCC-3′ |
| **Bpgm 5′-Forward** | 5′-AGACTAGGCAGGAGCTACAGCATAA-3′ |
| **Bpgm 5′-Reverse** | 5′-CACCTGCTGTTGGCAGGAATA-3′ |
| **Cre 3′-Forward** | 5′-CAGCAAAACCTGGCTGTGGATC-3′ |
| **Cre 3′- Reverse** | 5′-ATGAGCCACCATGTGGGTGTC-3′ |
| **Cre 5′-Forward** | 5′-TGGATGCCACCTCTGATGAAGTC-3′ |
| **Cre 5′-Reverse** | 5′-TCCTGGCATCTGTCAGAGTTCTCC-3′ |

Supplementary Table 2. Primer ~~and siRNA~~ sequences used in this study

| Primer (qRT-PCR) | Forward (5'-3') | Reverse (5'-3') |
| --- | --- | --- |
| *BPGM* (human) | ATCAGAAACTCAACAGCGAAGG | TGTGAATGGACCGATTAAGGAC |
| *BPGM* (mouse) | GAGCGTCACTATGGAGCCTT | AGTGGGCAGAGTGATGTTGA |
| *RET* (human) | ACAGGGGATGCAGTATCTGG | CCTGGCTCCTCTTCACGTAG |
| *MRC-1* (human) | GGCGGTGACCTCACAAGTAT | TTTTCATGGCTTGGTTCTCC |
| *CD163* (human) | ATCAACCCTGCATCTTTAGACA | CTTGTTGTCACATGTGATCCAG |
| *ARG-1* (human) | GGACCTGCCCTTTGCTGACATC | TCTTCTTGACTTCTGCCACCTTGC |
| *Il-10* (human) | GACTTTAAGGGTTACCTGGGTTG | TCACATGCGCCTTGATGTCTG |
| *β-actin* (human) | CATCCTCACCCTGAAGTACCCC | AGCCTGGATGCAACGTACATG |
| *β-actin* (mouse) | CAGCTTCTTTGCAGCTCCTT | CACGATGGAGGGAATACAG |

Supplementary Table 3. Primary antibodies for WB, IHC and IF used in this study

| **Antibody** | **WB** | **IHC** | **IF** | **Company** | **Cat No.** |
| --- | --- | --- | --- | --- | --- |
| **anti-BPGM** | 1:1000 | 1:500 |  | Proteintech | 17173-1-AP |
| **anti-RET** | 1:1000 | - |  | Abcam | ab134100 |
| **anti-Pan Kla** | 1:1000 | - |  | Jingjie | PTM-1401RM |
| **anti-ubiquitin** | 1:1000 | - |  | Jingjie | PTM-1106RM |
| **anti-P300** | 1:1000 | - |  | Proteintech | 20695-1-AP |
| **anti-CD206**  **anti-Ki67**  **anti-αSMA** | 1:1000  -  - | -  1:400  1:400 |  | Proteintech  Servicebio  Servicebio | 18704-1-AP  GB111499  GB111364 |
| **anti-CD68** |  |  | 1:200 | Servicebio | GB153109 |
| **anti-CD206** |  |  | 1:200 | Servicebio | GB113497 |
| **anti-CD86** |  |  | 1:200 | Servicebio | GB150054 |

Supplementary Table 4. siRNA sequences used in this study

| **Primer (qRT-PCR)** | **sense (5'-3')** | **antisense (5'-3')** |
| --- | --- | --- |
| ***BPGM*-homo-1** | GGAAGCAACUCAAAGCGUUTT | AACGCUUUGAGUUGCUUCCTT |
| ***BPGM*-homo-2** | GACCGGAGGUAUAAAGUAUTT | AUACUUUAUACCUCCGGUCTT |
| ***BPGM*-homo-3** | GCAGAUGGCUUUGAAUCAUTT | AUGAUUCAAAGCCAUCUGCTT |
| ***EP300*-homo-1** | GUCCUGGAUUAGGUUUGAUTT | AUCAAACCUAAUCCAGGACTT |
| ***EP300*-homo-2** | GGACUACCCUAUCAAGUAATT | UUACUUGAUAGGGUAGUCCTT |
| ***EP300*-homo-3** | CAUCACGGGUAUACAAAUATT | UAUUUGUAUACCCGUGAUGTT |
| ***RET*-homo** | GGCUCUUCAACCUUCUGAATT | UUCAGAAGGUUGAAGAGCCTT |

Supplementary Table 5. The fluorophore‑conjugated antibodies used in flow cytometry

| **Antibody** | **Concentration** | **Company** | **Cat No.** |
| --- | --- | --- | --- |
| **FITC-anti human CD206 antibody** | 1:50 | Biolegend | 321103 |
| **Brilliant Violet anti human CD68 antibody** | 1:50 | Biolegend | 333827 |
